# Presynaptic dystrophic neurites surrounding amyloid plaques are sites of microtubule disruption, BACE1 elevation, and increased Aβ generation in Alzheimer’s disease

**DOI:** 10.1007/s00401-016-1558-9

**Published:** 2016-03-18

**Authors:** Katherine R. Sadleir, Patty C. Kandalepas, Virginie Buggia-Prévot, Daniel A. Nicholson, Gopal Thinakaran, Robert Vassar

**Affiliations:** Department of Cell and Molecular Biology, Feinberg School of Medicine, Northwestern University, Chicago, IL 60611 USA; Departments of Neurobiology, Neurology and Pathology, The University of Chicago, Chicago, IL 60637 USA; Department of Neurological Sciences, Rush University, Chicago, IL 60612 USA; The Neurodegeneration Consortium, Institute for Applied Cancer Science (IACS), The University of Texas MD Anderson Cancer Center, Houston, TX 77054 USA

**Keywords:** Alzheimer’s disease, Amyloid, Aβ, BACE1, Dystrophic neurite, Microtubule, Tubulin, Axonal transport, Cathepsin D, 5XFAD mice

## Abstract

Alzheimer’s disease (AD) is characterized by amyloid plaques composed of the β-amyloid (Aβ) peptide surrounded by swollen presynaptic dystrophic neurites consisting of dysfunctional axons and terminals that accumulate the β-site amyloid precursor protein (APP) cleaving enzyme (BACE1) required for Aβ generation. The cellular and molecular mechanisms that govern presynaptic dystrophic neurite formation are unclear, and elucidating these processes may lead to novel AD therapeutic strategies. Previous studies suggest Aβ may disrupt microtubules, which we hypothesize have a critical role in the development of presynaptic dystrophies. To investigate this further, here we have assessed the effects of Aβ, particularly neurotoxic Aβ42, on microtubules during the formation of presynaptic dystrophic neurites in vitro and in vivo. Live-cell imaging of primary neurons revealed that exposure to Aβ42 oligomers caused varicose and beaded neurites with extensive microtubule disruption, and inhibited anterograde and retrograde trafficking. In brain sections from AD patients and the 5XFAD transgenic mouse model of amyloid pathology, dystrophic neurite halos with BACE1 elevation around amyloid plaques exhibited aberrant tubulin accumulations or voids. At the ultrastructural level, peri-plaque dystrophies were strikingly devoid of microtubules and replete with multi-lamellar vesicles resembling autophagic intermediates. Proteins of the microtubule motors, kinesin and dynein, and other neuronal proteins were aberrantly localized in peri-plaque dystrophies. Inactive pro-cathepsin D also accumulated in peri-plaque dystrophies, indicating reduced lysosomal function. Most importantly, BACE1 accumulation in peri-plaque dystrophies caused increased BACE1 cleavage of APP and Aβ generation. Our study supports the hypothesis that Aβ induces microtubule disruption in presynaptic dystrophic neurites that surround plaques, thus impairing axonal transport and leading to accumulation of BACE1 and exacerbation of amyloid pathology in AD.

**Electronic supplementary material:**

The online version of this article (doi:10.1007/s00401-016-1558-9) contains supplementary material, which is available to authorized users.

## Introduction

The β-amyloid (Aβ) peptide, the major component of amyloid plaques, has a crucial early role in Alzheimer’s disease (AD) pathogenesis [58]. The membrane aspartic protease β-site amyloid precursor protein (APP) cleaving enzyme 1 (BACE1) is the major β-secretase enzyme that initiates the production of Aβ from APP [20, 54, 66, 79]. Mutations in APP at the BACE1 cleavage site cause (K670N/M671L [38], A673V [8]) or prevent (A673T [24]) AD by increasing or decreasing BACE1 cleavage of APP and Aβ production, respectively. Also, a mutation at the β′-site (E682K [87]) causes AD by shifting BACE1 cleavage toward β-site processing of APP and Aβ generation. These mutations and other evidence strongly support therapeutic BACE1 inhibition for AD [67]. BACE1 inhibitor drugs are in clinical trials; however, *the safety and efficacy of these drugs are unknown*. BACE1 null mice have multiple neurological phenotypes indicating BACE1 inhibitor drugs could have mechanism-based toxicities [67]. BACE1 levels are elevated in AD brain [14, 18, 31, 80, 86] potentially necessitating high doses of BACE1 inhibitor drugs, thus increasing the risk of side effects. *Lowering/normalizing BACE1 levels in AD brain rather than direct inhibition of enzyme activity offers an alternative therapeutic approach that could avoid BACE1 inhibitor side effects.* Elucidating the mechanism of BACE1 elevation in AD is essential for developing BACE1 lowering strategies.

While the mechanism of BACE1 elevation in brains of AD patients or mouse models of AD is not yet clear, recent data indicate that BACE1 levels are upregulated during stresses associated with AD risk, such as energy deprivation [39, 68], hypoxia and stroke [56, 71, 84], oxidative stress [61], and traumatic brain injury [2, 65]. A large number of molecular pathways have been proposed to increase BACE1 levels: increased caspase 3 activity leading to impaired lysosomal degradation [26, 60], Cdk5 phosphorylation of transcription factor Stat3 [72], altered microRNAs [3, 12, 17, 69, 88], transcription factor HIF1α activity, [84], elevated phosphorylation of the elongation initiation factor eIF2α [39]. Thus, BACE1 appears to be a stress-response protein that can be regulated via diverse molecular pathways, making it challenging to identify the precise mechanism(s) involved in AD-relevant BACE1 elevation.

Insights into the mechanism of BACE1 elevation in AD have come from analysis of the localization pattern of increased BACE1 in the brains of AD patients and transgenic mouse models of amyloid pathology. Importantly, BACE1 elevation is not uniform throughout the brain, but is concentrated in presynaptic dystrophic neurites that surround amyloid plaques [25, 86] where it potentially spurs Aβ generation and plaque growth. Close proximity to plaques is associated with presynaptic dystrophy and Aβ42 oligomers increase BACE1 levels in cultured neurons [48, 49], thus implicating Aβ neurotoxicity in these processes. Reticulon 3 is involved in dystrophic neurite formation [19, 52, 53], but the role of Aβ is poorly understood. We recently determined that BACE1-YFP expressed from a doxycycline-inducible transgene lacking the endogenous 5′ UTR that controls BACE1 translation accumulates around plaques in an APP transgenic mouse similar to that observed in AD [48]. These results suggest that BACE1 elevation in AD occurs via a post-translational mechanism involving Aβ neurotoxicity that is closely associated with amyloid plaques, and does not appear to involve transcriptional or translational regulation.

Here, we show by live-cell imaging that Aβ42 oligomers cause microtubule disruption and neuritic beading. In BACE1-positive dystrophic neurites surrounding amyloid plaques of AD and the 5XFAD transgenic mouse model, tubulin isoforms are mis-localized, often forming aberrant accumulations or voids. By EM, 5XFAD dystrophic axons appear distended with multi-lamellar vesicles, but notably lack intact microtubules. This observation, together with aberrant localization of microtubule motor proteins and other neuronal proteins, and evidence of reduced lysosomal function and autophagic intermediate accumulation, suggests that microtubule-based transport is impaired in dystrophic neurites surrounding amyloid plaques. Most importantly, BACE1 and APP accumulate in peri-plaque dystrophies to very high levels and lead to increased generation of BACE1-cleaved APP products, including Aβ42 that may exacerbate plaque growth. Taken together, our results suggest that amyloid plaques cause a local toxic effect, possibly mediated by soluble Aβ42 oligomers, that generates presynaptic dystrophic neurites by disrupting microtubles and impairing transport. As a result, peri-plaque dystrophies accumulate BACE1, APP, and γ-secretase, further contributing to Aβ generation and plaque growth in a feed-forward mechanism.

## Materials and methods

### Primary neuron culture, Aβ42 oligomer preparation, and immunofluorescence

E15.5-16.5 C57BL/6 mouse cortical neurons were plated on poly-l-lysine (Sigma) coverslips in 12-well plates (150,000 cells/well) in neurobasal media supplemented with 2 % B-27, 500 μM glutamine, 10 % horse serum, and 2.5 μM glutamate. After 2 h, medium was changed to neurobasal with 2 % B-27, 500 μM glutamine, and 2.5 μM glutamate. After 1–3 days, medium was replaced with neurobasal plus 2 % B-27 and 500 μM glutamine. Following 9 days in culture, neurons were treated with 1 μM Aβ42 oligomers or vehicle prepared as previously described [48, 55]. Briefly, lyophilized recombinant Aβ42 peptide (rPeptide, cat# A-1163-1) was HFIP treated, dried down, then resuspended to 5 mM in dry DMSO (Molecular Probes, # D12345) and brought to 100 μM in cold, 4 mM HEPES pH 8, incubated on ice at 4 °C for 24 h to generate oligomers. For control cultures, DMSO alone was added to 4 mM HEPES pH 8 and incubated as described. After 72 h of Aβ42 treatment, neurons were fixed for 20 min in 4 % paraformaldehyde, 0.12 M sucrose in PBS, permeabilized in 0.5 % Triton, and incubated in anti-βIII tubulin mouse monoclonal antibody (TuJ1, gift of Dr. Lester Binder, 1:500) followed by donkey anti-mouse Alexa 568-conjugated secondary antibody (Molecular Probes, 1:1000) and counterstained with 300 nM DAPI. Coverslips were mounted using Prolong Gold (Molecular Probes), and images acquired on a Nikon A1 confocal microscope with a 60× objective (NA 1.4) and NIS Elements software.

### Live imaging of neurons

Primary neurons prepared as above were plated (150,000 cells/dish) in 35-mm glass-bottom culture dishes (MatTek # P35G-1.5-14C). After 11–12 days, medium was replaced with neurobasal containing 250 nM of Tubulin Tracker (Molecular Probes #T34075), neurons incubated for 30 min at 37 °C, rinsed twice with warmed media, and original media replaced. 10 μM Aβ42 oligomers or vehicle, prepared as above, were added and neurons imaged on an Andor Spinning Disk confocal microscope with a 60× objective (NA 1.49). Imaging began within 30 min of treatment and continued for 3 h, with a 100-ms exposure taken every 5 min, or 6 h, with a 100-ms exposure taken every hour, at 20–25 locations on the coverslip. Images were captured using Metamorph and intensity quantified in ImageJ. Statistical analysis was done using a two-tailed *t* test in Prism.

### Mice

5XFAD mice were generated as previously described [40] and maintained by crossing transgene positive males with B6/SJL F1 hybrid females (Jackson Laboratories). As negative controls, 5XFAD mice were crossed to BACE1^−/−^ mice [4] to generate 5XFAD;BACE1−/− mice. All animal work was done in accordance with Northwestern University IACUC approval.

### Human tissue immunofluorescence

Human post-mortem brain tissue was obtained from three AD patients and three cognitively normal controls (Supplementary Table S2) diagnosed at the Cognitive Neurology and Alzheimer’s Disease Center with approval from the Northwestern University IRB. 40-μm floating sections of superior temporal gyrus were stained and imaged as described for murine tissue, except that primary and secondary antibody incubations were 48 h at 4 °C to improve antibody penetration, methoxy XO4 was added after secondary antibody incubation to label plaques, and 0.2 % Sudan Black in 40 % ethanol was used to quench autofluorescence. Sections incubated in parallel without primary antibody were included as negative controls for autofluorescence and background binding of secondary antibody. A minimum of 10 plaques per case were imaged and analyzed on a Nikon A1 confocal microscope with a 60× objective (NA 1.4) and NIS Elements software. Image acquisition settings were maintained the same between AD and control cases. We note that the βIII-tubulin antibody TuJ1 is very well characterized and produced the expected βIII-tubulin staining pattern in cognitively normal controls (not shown) and in normal-appearing neuropil in AD cases (Fig. 3a, c), which served as positive controls. The no-primary antibody negative control produced only weak non-specific background staining in control and AD brain sections (not shown).

### Murine tissue immunofluorescence

Mice were perfused with ice-cold PBS containing protease and phosphatase inhibitors (Calbiochem), brains harvested, and one hemibrain/mouse drop fixed in 4 % paraformaldehyde/PBS and cryopreserved in 30 % w/v sucrose/PBS for sectioning. The other hemibrain/mouse was dissected into cortex and hippocampus and flash frozen in LN2 for biochemistry. 30-μm coronal or sagittal floating brain sections were cut and subjected to antigen retrieval for 1 h at 80 °C in 0.1 M sodium citrate pH 9 followed by 16 mM glycine in Tris-Buffered Saline with 0.25 % triton-X 100 (TBS-T) and blocked in 5 % donkey serum in TBS-T. Sections were incubated overnight at 4 °C with primary antibodies listed in Supplementary Table 1, followed by secondary antibodies (donkey anti-mouse, rabbit or goat conjugated to Alexa 488, 568 or 647; Molecular Probes) used at the same concentration as primary antibody for 2–3 h at room temperature (except 4 °C overnight to improve penetration in the case of anti-tubulin antibodies) plus 300 nM DAPI. Sections incubated in parallel without primary antibody were included as negative controls for autofluorescence and background binding of secondary antibody. Sections were mounted with Prolong Gold (Molecular Probes) and images acquired on a Nikon A1 confocal microscope with a 60× objective (NA 1.4) and NIS Elements software. All image acquisition settings were maintained the same between genotypes (5XFAD;BACE1+/+ and 5XFAD;BACE1−/−). For all proteins whose plaque-associated co-localization with BACE1 was not previously published, 5–8 male and female 5XFAD mice at 5–6 or 9 months of age (Table S3) were analyzed, and 20–40 plaques were imaged for each antibody stain. For co-localization patterns of proteins with BACE1 that have been previously published (Table S4), we did not include additional mice but did confirm the originally reported staining patterns for βIII-tubulin (Fig. 3), MAP2 (Fig. 6), synaptophysin (Fig. 6), APP (Figs. 8, 9), and Aβ/3D6 (Figs. 8, 9). We validated neoepitope antibodies to APP cleavage products using brain sections from 5XFAD;BACE1−/− mice (negative control). These sections were processed and imaged in parallel with brain sections from 5XFAD;BACE1+/+ mice. For other antibodies, we considered normal-appearing neuropil regions distant from plaques to be an internal positive control, as well as normal-appearing regions of the stratum lucidum (Fig. 6, bottom row), which serves as a positive control for normal BACE1 localization and co-localization with other proteins.

### Electron microscopy

5XFAD mouse brain tissue was prepared for electron microscopy as previously described [25]. Briefly, mice were anesthetized with isofluorane (Isothesia, Butler), perfused transcardially with 0.12 M PBS (pH 7.4) for 1 min, then a dilute aldehyde mixture (1 % paraformaldehyde, 1.25 % glutaraldehyde, 0.02 mM CaCl_2_ in 0.1 M sodium cacodylate buffer) for 30 min, and a concentrated aldehyde mixture (2 % paraformaldehyde, 2.5 % glutaraldehyde, 0.04 mM CaCl_2_ in 0.1 M sodium cacodylate buffer) for 10 min. The brain was removed and placed in ice-cold concentrated fixative on a shaker at 4 °C overnight. The following day brain was bisected, rinsed 3× 20 min in 0.12 M TBS and cut into 70 μm coronal sections. Sections were washed in 0.12 M phosphate buffer (PB) 3× 10 min at 4 °C, treated with 2 % OsO_4_ in 0.12 M PB for 1 h at 4 °C, and washed 3× 10 min in 0.12 M PB. The tissue was then dehydrated in graded ethanols and propylene oxide, infiltrated with 1:1 araldite:propylene oxide, flat embedded between aclar sheets and cured for 48 h at 60 °C. Regions of interest were subdissected and re-embedded as above. Serial ultrathin sections (65 nm) were cut, placed onto formvar-coated slotted grids, then stained with 5 % aqueous uranyl acetate for 15 min, followed by Reynold’s lead citrate (1.33 g lead nitrate, 1.76 g sodium citrate, 30 ml distilled water, 8 ml of 1 N NaOH, then diluted to 50 ml) for 10 min, and rinsed in ultrapure dH_2_O. Images (7500–20,000×) were acquired on a JEOL 1200EX electron microscope (JEOL Ltd., IL, USA) from 10 to 30 serial sections. ImageJ software was used for processing image stacks of serial sections.

### Immunoblotting

Frozen brain tissue was homogenized in RIPA buffer and 20 μg of homogenate separated by 10 % tris–glycine SDS-PAGE. Protein was transferred onto 0.45 μm PVDF membrane, stained with 0.1 % Ponceau, and imaged. Blots were incubated with anti-APP antibody (6E10, Covance Sig-39300 1:2000) or anti-cathepsin D (Abcam # ab75852 1:1000) followed by HRP-conjugated anti-mouse or anti-rabbit secondary antibody (Vector Laboratories 1:10,000). Blots were visualized using chemiluminescence (Luminata Crescendo, Millipore), signals quantified using a Kodak Image Station 4000 R, normalized to Ponceau, densitometric analyses performed using Kodak 1D 3.6 image analysis software, and statistics analyzed in Prism GraphPad.

### Statistical analyses

Statistical differences for immunoblot experiments were determined using two-tailed Student’s *t* test (GraphPad Prism Software, Inc., San Diego, CA, USA). Graphed data are presented as the mean ± SEM, and *p* < 0.05 was considered significant.

## Results

### Aβ42 causes neuritic beading and microtubule disruption in primary neurons in vitro

**Fig. 1:**
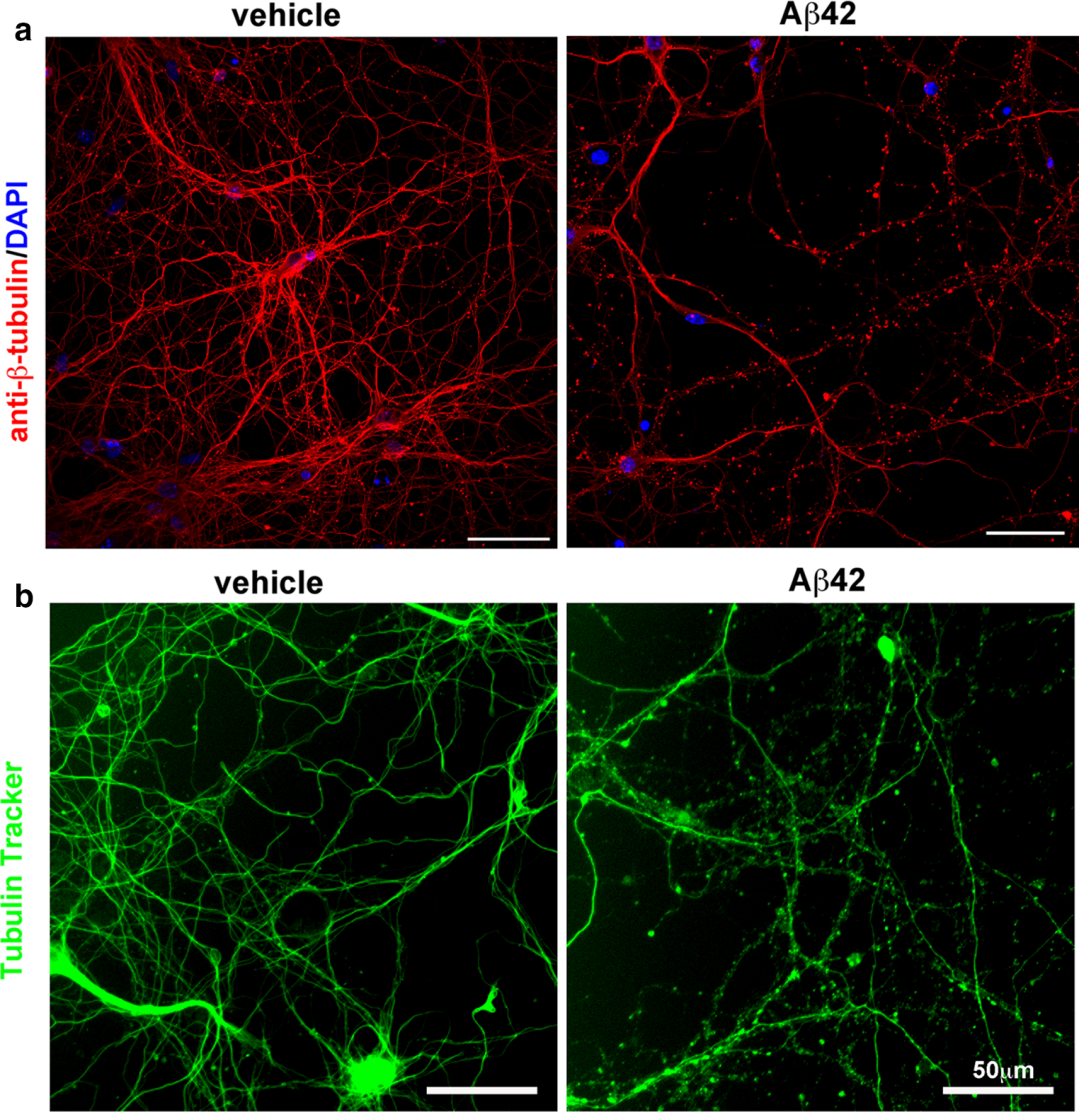
Oligomeric Aβ42 causes neurite beading and microtubule disruption in primary neurons. **a** Primary cortical neurons were cultured from e15.5 mouse embryos and after 9 days in vitro were exposed to 1 μM Aβ42 oligomers or vehicle. After 72 h, coverslips were fixed in 4 % paraformaldehyde, permeabilized, and stained with the antibody TuJ1 against βIII-tubulin (*red*) and DAPI (*blue*). Neurite beading is very prominent in Aβ42-treated cultures, while neurites appear smooth and unbeaded in vehicle-treated neurons. **b** Primary neurons were isolated as described in **a**, and after 12 days in vitro were exposed to 10 μM Aβ42 oligomers or vehicle. After 22 h of Aβ42 treatment, the fluorescent microtubule probe Tubulin Tracker was added to a final concentration of 250 nM for 30 min. Unfixed, live neurons were then imaged at ×20 using a Nikon Eclipse TS100 microscope with NIS Elements software. Note that Aβ42-induced neurite beading visualized by Tubulin Tracker appears very similar to that observed with βIII-tubulin immunostaining in **a**, and occurs more rapidly with higher Aβ42 concentration. Taken together, these results strongly suggest that Aβ42 disrupts the organization of microtubules in neurons. *Scale bars* in all frames 50 μm

In addition to amyloid plaques and neurofibrillary tangles, other hallmark lesions of the Alzheimer’s disease brain include swollen, dystrophic neurites that surround amyloid plaques (reviewed in [9, 51]). Since these dystrophic neurites are predominantly found in very close proximity to, and often in contact with, plaques [25, 62] a neurotoxic form of Aβ (e.g., oligomers) likely causes dystrophy formation. To test this hypothesis, we isolated primary cortical neurons from embryonic mice and exposed them to 1 μM of oligomeric Aβ42 for 72 h, then performed immunofluorescence microscopy for neuron-specific βIII-tubulin. Aβ42-treated neurons displayed processes with numerous βIII-tubulin accumulations along their lengths resembling beads on a string, compared to vehicle-treated neurons (Fig. 1a). Neuritic beading was not likely associated with processes related to cell death caused by high concentrations of Aβ42, since we previously observed that 5 days of treatment with 1 and 2 μM Aβ42 oligomers did not increase caspase 3 cleavages in primary neurons [49]. These beaded structures bore a striking resemblance to tubulin accumulations observed in dystrophic neurites around plaques in vivo (Fig. 3), suggesting a similar mechanism of generation. Our observation of tubulin accumulation in beaded processes in Aβ42-treated cultured neurons is in agreement with extreme beading of primary neurons following 4 days of exposure to 15 μM Aβ42 [11].

Neuritic dystrophy and beading are observed in neurons exposed to a variety of stressors and may be part of a regulated neurite degeneration pathway separate from cellular apoptosis [10, 21, 28]. The presence of abnormal βIII-tubulin accumulations in Aβ42-treated primary neurons suggested that Aβ42 could disrupt microtubule networks, and that impaired axonal transport could be a specific and early effect of Aβ42 leading to serious neuronal and synaptic dysfunction. To test this hypothesis, we followed microtubule dynamics in real time by live-cell imaging to determine if and when Aβ42 could cause microtubule disruption. We incubated primary neurons with Tubulin Tracker (Oregon Green-labeled taxol) that binds only to polymerized tubulin in microtubules, and then exposed the neurons to 10 μM Aβ42 oligomers (Fig. 1b) to model the potentially high Aβ42 oligomer concentrations in the immediate vicinity of amyloid deposits [29]. After 22 h of treatment, neuritic beading that strongly resembled the previously observed aberrant βIII-tubulin immunostaining pattern (Fig. 1a) was apparent in live Aβ42-treated Tubulin Tracker-labeled neuron cultures (Fig. 1b), while vehicle-treated neurons remained healthy with mostly smooth processes. These findings suggested that Aβ42 exposure results in microtubule disruption leading to abnormal tubulin accumulation and neuritic beading in neurons.

**Fig. 2:**
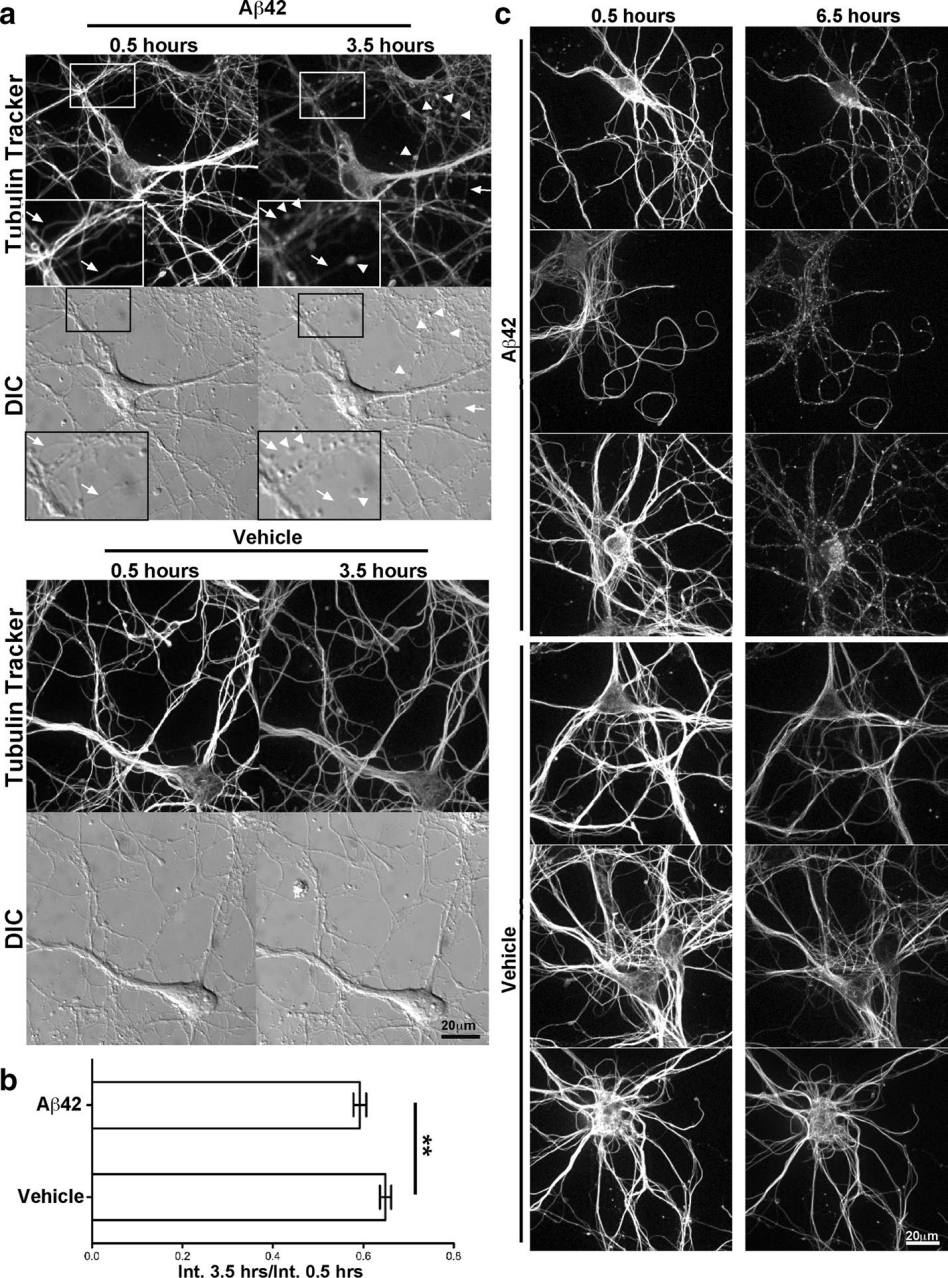
Microtubule disruption in primary neurons is an early response to oligomeric Aβ42. Primary cortical neurons were cultured as in Fig. 1, and after 12 days in vitro, were labeled for 30 min with 250 nM Tubulin Tracker, rinsed, and then exposed to 10 μM Aβ42 oligomers or vehicle. Live imaging on an Andor spinning disk confocal microscope began 30 min after addition of Aβ42. **a** Fluorescence and differential interference contrast (DIC) images were acquired every 5 min for 3 h. Neurite beading, microtubule accumulation in varicosities (*arrowheads*), and microtubule fragmentation (*arrows*) in neurites were present 3.5 h after Aβ42 addition, in the absence of significant cell death or BACE1 elevation [49]. In contrast, the morphology of vehicle-treated neurons was unaffected, with the exception of a moderate decrease of fluorescence intensity. DIC imaging revealed that neurites of Aβ42-treated neurons were intact and continuous, even though they exhibited beading and microtubule fragmentation, indicating that the observed microtubule disruption was not the result of physical degeneration of neurites. *Insets* show higher magnification images of the boxed region of the Aβ42-treated culture to accentuate varicosity formation and microtubule fragmentation. **b** The ratios of Tubulin Tracker fluorescence intensity (Int.) at 3.5 h (hours) to that at 0.5 h after Aβ42 treatment were calculated for 46 image fields from two separate experiments and averaged for Aβ42-treated and vehicle-treated neurons. Overall Tubulin Tracker fluorescence intensity decreased in neurons over time due to photo-bleaching, as indicated by fluorescence intensity ratios below 1. Nevertheless, the Tubulin Tracker fluorescence intensity ratio for Aβ42-treated neurons showed a small but highly significant decrease compared to vehicle treatment (*p* = 0.004), suggesting microtubule depolymerization and reduced stability of microtubule networks after only 3.5 h of Aβ42 exposure*. Error bars* SEM; **, *p* < 0.01. **c** To investigate longer Aβ42 treatment times and minimize photo-bleaching, primary neuron cultures were prepared as above and images collected once every 60 min for 6 h. Neurite beading, aberrant microtubule localization, and microtubule fragmentation were even more prevalent and pronounced in neurons after 6.5 h compared to 3.5 h of Aβ42 treatment, while vehicle-treated neurites continued to appear normal. *Scale bars* 20 μm for all images in **a**, **c**

To determine how early microtubule disruption and neuritic beading occur after exposure to Aβ42, we used spinning disk confocal microscopy to image live Tubulin Tracker-labeled primary neurons continuously every 5 min after 10 μM Aβ42 oligomer or vehicle treatment. We observed that noticeable microtubule disruption and beading occurred on neurites of primary neurons as early as 3.5 h following Aβ42 treatment, while vehicle-treated cultures appeared normal (Figs. 2a, S1). The very early appearance of disrupted microtubules in beaded neurites suggested that this is one of the primary ways in which Aβ42 oligomers could impair neuronal function. Also, differential interference contrast microscopy indicated that most of the neurites with beads or aberrant microtubule organization were still continuous and intact, although morphologically abnormal (Figs. 2a, S1). These results were highly reproducible, as they were representative of six individual experiments, all performed using separate primary neuron and Aβ42 preparations. We also observed a greater overall decrease in Tubulin Tracker fluorescence after 3 h in the Aβ42-treated neurons compared to vehicle (Fig. 2b), suggesting that Aβ42 causes microtubule depolymerization. To limit photodamage, we performed an identical live-imaging experiment, except capturing an image once every hour for 6 h, and observed even more striking neuritic beading and microtubule disruption in Aβ42-treated neurons, while vehicle-treated neurons showed very little change (Fig. 2c).

Aβ42 oligomer concentrations in the range of 1–10 μM induce cell death in primary neuron culture (reviewed in [16]). To determine whether neuritic beading and microtubule disruption were the results of Aβ42-induced cell death processes, we repeated Tubulin Tracker labeling of primary neurons followed by Aβ42 treatment for 3.5 h, then added propidium iodide to label the nuclei of dead cells (Fig. S1). Although we noted Aβ42-induced neuritic beading and microtubule disruption (Fig. S1a), the percentage of live cells as measured by absence of propidium iodide uptake was identical for both Aβ42-treated and vehicle control neurons (Fig. S1b). This result demonstrates that neuritic beading and microtubule disruption precede the induction of cell death in Aβ42-treated primary neurons.

The Aβ42-induced reduction and aberrant spatial distribution of microtubules in neurites suggested that microtubule-based transport would be impaired in Aβ42-treated primary neurons. It has also been reported that trafficking of BDNF [44] mitochondria [70], and other axonal cargo [57] is impaired by Aβ, so we hypothesized BACE1 transport may be affected as well. To test this hypothesis, we transfected primary neurons with BACE1-YFP and neuropeptide Y-mCherry (NPY-mCherry) fusion constructs, treated with 10 μM Aβ42 oligomers or vehicle, and performed live-cell imaging and kymograph analysis to assess vesicular movement in neurites. We observed that the proportion of motile BACE1-YFP and NPY-mCherry puncta was decreased in neurites of Aβ42-treated neurons, compared to vehicle (Fig. S2). We infer that the effect of Aβ42 on trafficking in neurites is not limited to BACE1-containing vesicles, as the movement of NPY-mCherry puncta was also decreased. Taken together, our results suggest that Aβ42 causes microtubule depolymerization and network disruption, leading to neuritic beading and impaired microtubule-based axonal transport in neurons.

### Tubulin is aberrantly localized and microtubules are disrupted in peri-plaque presynaptic dystrophic neurites in vivo

**Fig. 3:**
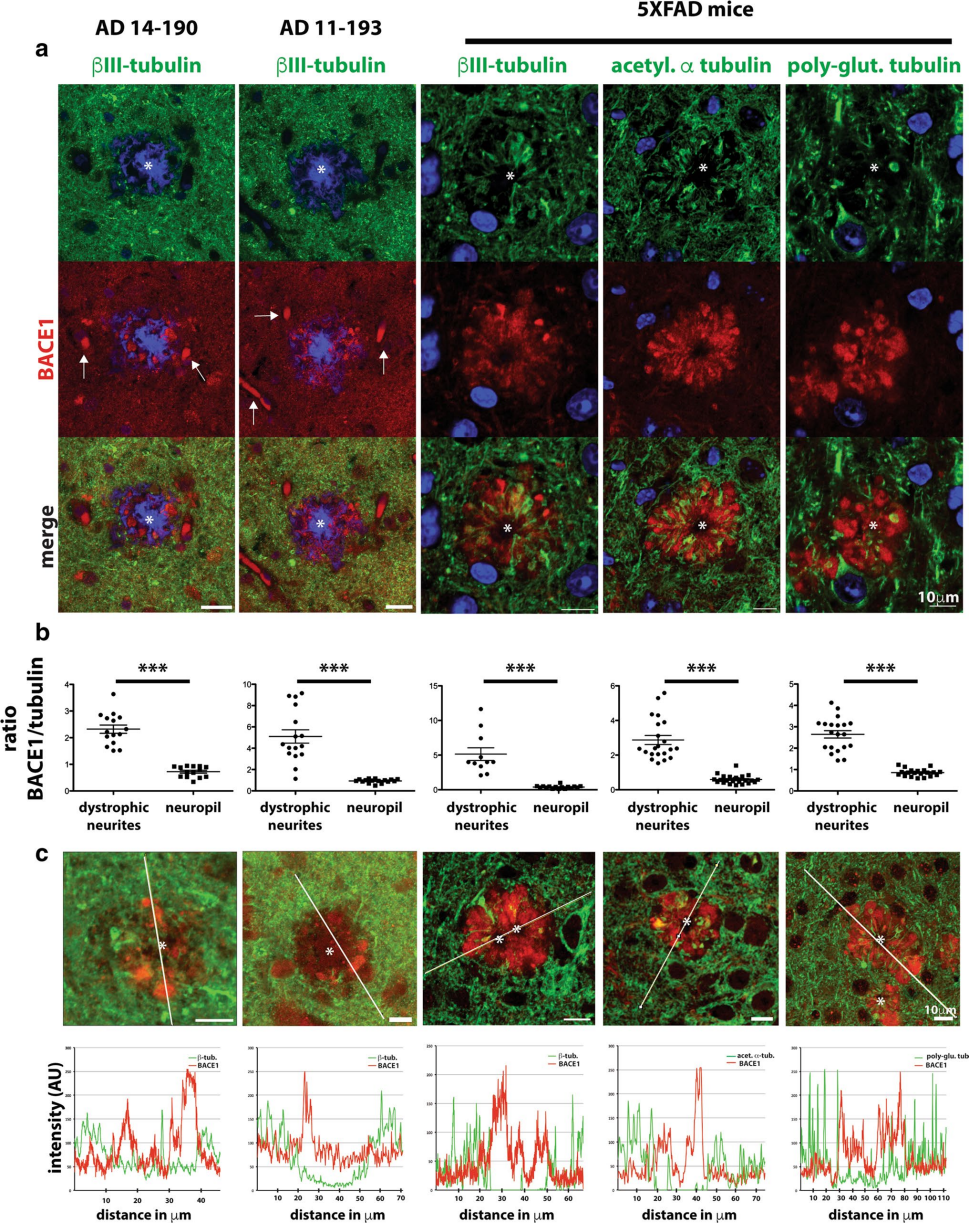
Tubulin isoforms are aberrantly localized in BACE1-positive peri-plaque dystrophic neurites in vivo. **a** Post-mortem brain sections of superior frontal gyrus from three AD patients and three cognitively normal controls were stained with antibodies against BACE1 (*red*) and βIII-tubulin (*green*), and methoxy XO4 (*blue*) to label amyloid deposits. Representative plaques from two cases (AD 14–190 and AD 11–193, *Columns* 1–2) are shown; additional examples in Fig. S2. Cognitively normal controls were negative for plaques, as expected (not shown). As previously reported [86], BACE1 immunostaining was observed in a halo of dystrophic neurites surrounding individual plaques. In contrast, βIII-tubulin immunostaining was almost entirely absent in peri-plaque BACE1-positive dystrophies. Non-dystrophic neuropil in areas adjacent to plaques exhibited normal intensities and patterns of βIII-tubulin immunostaining. Some red fluorescence (*white arrows*) represents non-specific background from blood vessels. Brain sections from the aggressive amyloid 5XFAD mouse model were stained with antibodies against BACE1 (*red*) and βIII-tubulin, acetylated α-tubulin or polyglutamylated tubulin (*green*). Representative 5XFAD amyloid deposits are shown (*Columns* 3–5), with plaque cores marked by asterisks; additional examples in Fig. S2. Similar to human AD, BACE1 accumulated in peri-plaque dystrophic neurites in 5XFAD brain sections. Additionally, the different tubulin isoforms were generally reduced in dystrophic halos around plaques, although tubulins often occurred in amorphous accumulations that showed little co-localization with BACE1 immunostaining. *Scale bars* in all frames 10 μm. **b** Immunofluorescence intensities of BACE1 and tubulin isoforms (as indicated at *top of each column*) in peri-plaque dystrophic neurites were measured and BACE1/tubulin intensity ratios calculated and compared to those in normal-appearing regions of neuropil within the same frame (Supplementary Methods). For human tissue, 16 dystrophies and corresponding non-dystrophic regions were measured per case. For murine tissue, BACE1/tubulin isoform ratios were determined for 11–20 BACE1-positive dystrophic regions and a corresponding number of nearby neuropil areas. BACE1/tubulin ratios for AD 14–190 and AD 11–193 (*Panels* *1* and *2*, respectively) and 5XFAD mice (*Panels* *3*–*5*) are shown. While there was substantial variation for dystrophic neurites, BACE1/tubulin intensity ratios for dystrophies were significantly elevated compared to those for normal neuropil for both AD cases and 5XFAD mice. *Error bars* SEM; ***p < 0.001. **c** The quantitative relationships between BACE1 and tubulin isoform immunofluorescence intensities by another method, intensity profiles as a function of distance across the plaque were generated (Supplementary Methods). Representative plaques are shown (*upper row*) for AD 14–190 and AD 11–193 (*Panels 1* and *2*, respectively) and 5XFAD mice (*Panels* *3*–*5*). *White line* indicates path through plaque from which the intensity profiles were generated (*bottom row*). Note the inverse relationships between BACE1 and tubulin intensities as a function of distance across the plaque in each case. Taken together, these results suggest that tubulin, and hence microtubules, is reduced and/or mis-localized in peri-plaque dystrophic neurites. *AU* arbitrary units. *Scale bars* in all frames 10 μm

In both AD and APP transgenic mouse brains, BACE1 accumulates in swollen presynaptic dystrophic neurites that surround amyloid plaques in close proximity [25, 85, 86]. To gain insight into the mechanism of Aβ-induced BACE1 elevation in peri-plaque dystrophies and the potential role of microtubules, we performed immunofluorescence staining of superior temporal gyrus from AD patients and cognitively normal controls (Table S2) with antibodies against BACE1 and neuron-specific βIII-tubulin, as well as methoxy XO4 to label fibrillar amyloid deposits. As we have previously reported [25, 86], in AD brain BACE1 immunoreactivity was elevated in dystrophic neurites in a halo pattern immediately surrounding the methoxy XO4-positive amyloid plaque core. Interestingly, we observed a striking reduction of βIII-tubulin signal within the BACE1-positive peri-plaque halo, while the pattern of βIII-tubulin immunoreactivity seemed unaffected in nearby normal-appearing neuropil (Fig. 3a, first two columns; Fig. S3, first column) and in cognitively normal controls (not shown). To determine the quantitative relationships between BACE1 and βIII-tubulin immunoreactivities within the peri-plaque halo, we calculated the ratio of BACE1 to βIII-tubulin immunofluorescence intensities in dystrophic neurites (defined by high BACE1 signal) around plaques and in regions of normal neuropil distant from plaques for the AD cases (Fig. 3b). Importantly, we observed that the peri-plaque BACE1:βIII-tubulin ratio was significantly elevated (Fig. 3b, first two panels). We also generated intensity profiles of BACE1 and βIII-tubulin immunofluorescence signals through the plaque, and generally found an inverse relationship between BACE1 and βIII-tubulin intensities, such that high BACE1 signal typically occurred in areas of low βIII-tubulin intensity (Fig. 3c, first two columns). In cognitively normal controls, no plaques, and hence no areas of elevated BACE1 or loss of βIII-tubulin, were observed (not shown). These results confirm and extend our initial observation of decreased βIII-tubulin staining around plaques in AD [86]. Moreover, they suggest that reduced tubulin level, and hence low microtubule density, in dystrophic regions surrounding plaques is a pathologic feature of AD that is the consequence of short-range Aβ toxicity to neurites, and may have a role in BACE1 accumulation.

Post-mortem AD brain samples represent end-stage disease and thus provide limited information about pathogenesis. To gain insight into the development of Aβ-induced dystrophic neurites, we turned to our 5XFAD mouse model of amyloid pathology [40]. Our previous work showed that brains of AD patients and 5XFAD mice have elevated levels of BACE1 that accumulate in dystrophic presynaptic neuronal structures (likely axons and terminals) in a halo that surrounds the amyloid deposit [86]. Taken together, these and other data suggest that the 5XFAD mouse is a faithful model of BACE1 elevation in AD.

To assess the distributions of tubulin and BACE1 around amyloid deposits in our AD mouse model, we immunostained brain sections from five different 5- to 6-month-old 5XFAD mice with antibodies directed against specific isoforms of tubulin, including neuron-specific βIII-tubulin, acetylated α-tubulin, and polyglutamylated tubulin (Fig. 3a, third, fourth and fifth columns, respectively; Figs. S3, S4). BACE1 immunoreactivity was concentrated in a halo surrounding the core of the amyloid deposit, as previously reported [25, 85, 86]. Interestingly, similar to human AD, we observed that βIII-tubulin immunostaining was largely absent in BACE1-positive peri-plaque dystrophies. The βIII-tubulin signals that did occur in the peri-plaque halo appeared as amorphous accumulations with a staining pattern complementary to that of BACE1, i.e., regions of high βIII-tubulin intensity generally showed low BACE1 signal and vice versa. Acetylated and polyglutamylated isoforms of tubulin showed similarly reduced patterns of immunostaining in the peri-plaque halo. Additionally, we measured peri-plaque BACE1:tubulin ratios and intensity profiles for 5XFAD amyloid deposits (Fig. 3b, c) and found increased ratios and inverse relationships between BACE1 and tubulin isoforms, similar to our results for human AD plaques. We note that the peri-plaque human and mouse tubulin and BACE1 immunoreactivity patterns are similar but not identical, probably because mouse deposits are at most a few months old while human plaques are possibly many years old. At the end-stage of disease in post-mortem AD brain, there are very few, if any neurites near plaques that are not severely degenerated, thus potentially explaining the lower intensities of BACE1 and tubulin immunostaining in peri-plaque dystrophies of human AD compared to 5XFAD mouse.

**Fig. 4:**
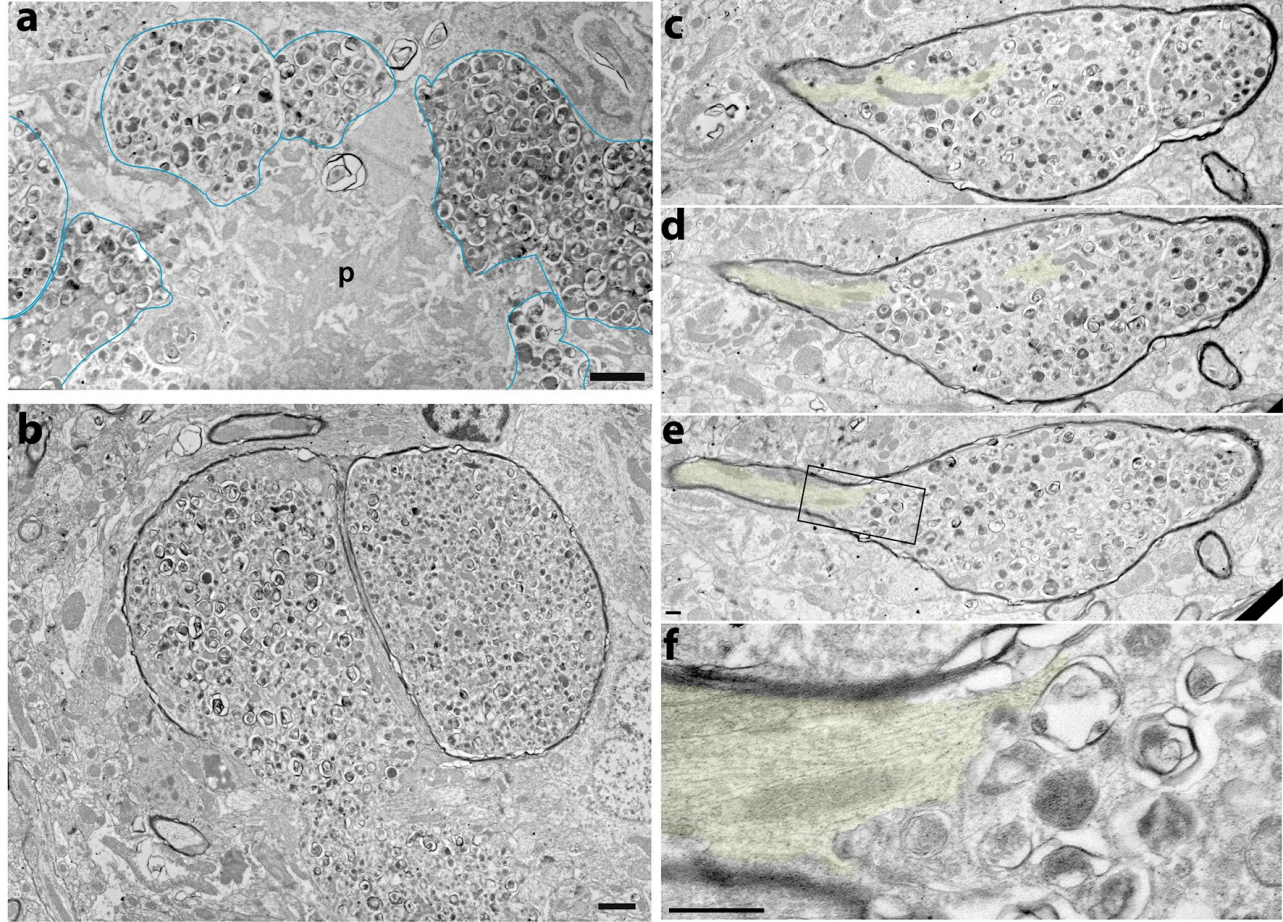
Microtubules are strikingly absent in peri-plaque dystrophic axons. 5XFAD brain tissue was prepared for electron microscopy (EM) and assessed ultrastructurally for the presence of microtubules in peri-plaque dystrophic axons. **a** As previously reported [25], electron microscopy of 5XFAD brain revealed amyloid plaques (p) surrounded by dystrophic neurites (representative examples outlined in *blue*) filled with electron-dense multi-lamellar vesicles, possibly autophagic intermediates. **b** EM of a transverse section through a pair of dystrophic myelinated axons filled with multi-lamellar vesicles. Note that the lower portion of the axon on the *left* has lost its myelin sheath. **c**–**f** EM of serial ultrathin sections occasionally revealed series of longitudinal sections through dystrophic axons near amyloid plaques. Shown is such a series of a peri-plaque dystrophic myelinated axon with a portion of normal axon appearing on the *left side* of the dystrophy. Intact, continuous microtubules (*yellow shading*) are present in the morphologically normal part of the axon, but then microtubules become disorganized and mostly disappear within the dystrophic region. Note the *dark outline* of the surrounding myelin sheath, definitively confirming axonal identity. These ultrastructural images provide direct evidence that microtubules are disrupted and largely absent in peri-plaque dystrophic regions of axons. Images are spaced ~200 nm apart. **f** Higher magnification of *rectangle* in **e**. *Scale bars* 1 μm

The reduction in tubulin isoforms in 5XFAD BACE1-positive dystrophies suggested that microtubules are decreased, disorganized, or disrupted in presynaptic dystrophic neurites near amyloid deposits. To investigate this possibility at an ultrastructural level, we examined ultrathin sections of 5XFAD mouse brain by electron microscopy (EM). We found swollen dystrophic neurites in abundance in close proximity or virtually contacting amyloid deposits (Fig. 4a). We confirmed the close physical association between BACE1-positive dystrophies and amyloid deposits in 3D reconstructions of multi-photon confocal microscopy images of live brain slices of BACE1-YFP;5XFAD multi-transgenic mice (Supplementary Text; Fig. S5, Videos S1, S2). By BACE1 immuno-EM, we previously reported that dystrophies surrounding plaques are engorged with large BACE1-negative electron-dense multi-lamellar vesicles and smaller BACE1-positive electron-translucent vesicles [25]. Here, by EM we found dystrophies that were definitively identified as axons, as indicated by the presence of myelin sheaths (Fig. 4b–f). Importantly, we found occasional EM series displaying normal-appearing axons with dense microtubule bundles that enter a dystrophic region within which microtubules are strikingly absent, reduced in length, or dramatically disorganized (Fig. 4c–f). These data suggest that in human AD and in amyloid pathology mouse models, peri-plaque dystrophic axons are deficient in normal functional microtubule networks for axonal transport. Vesicles may enter but cannot transit or exit dystrophic regions due to absence of microtubules, which could explain the dramatic accumulation of BACE1 around plaques.

### Microtubule motor proteins are aberrantly localized in BACE1-positive peri-plaque dystrophic neurites

**Fig. 5:**
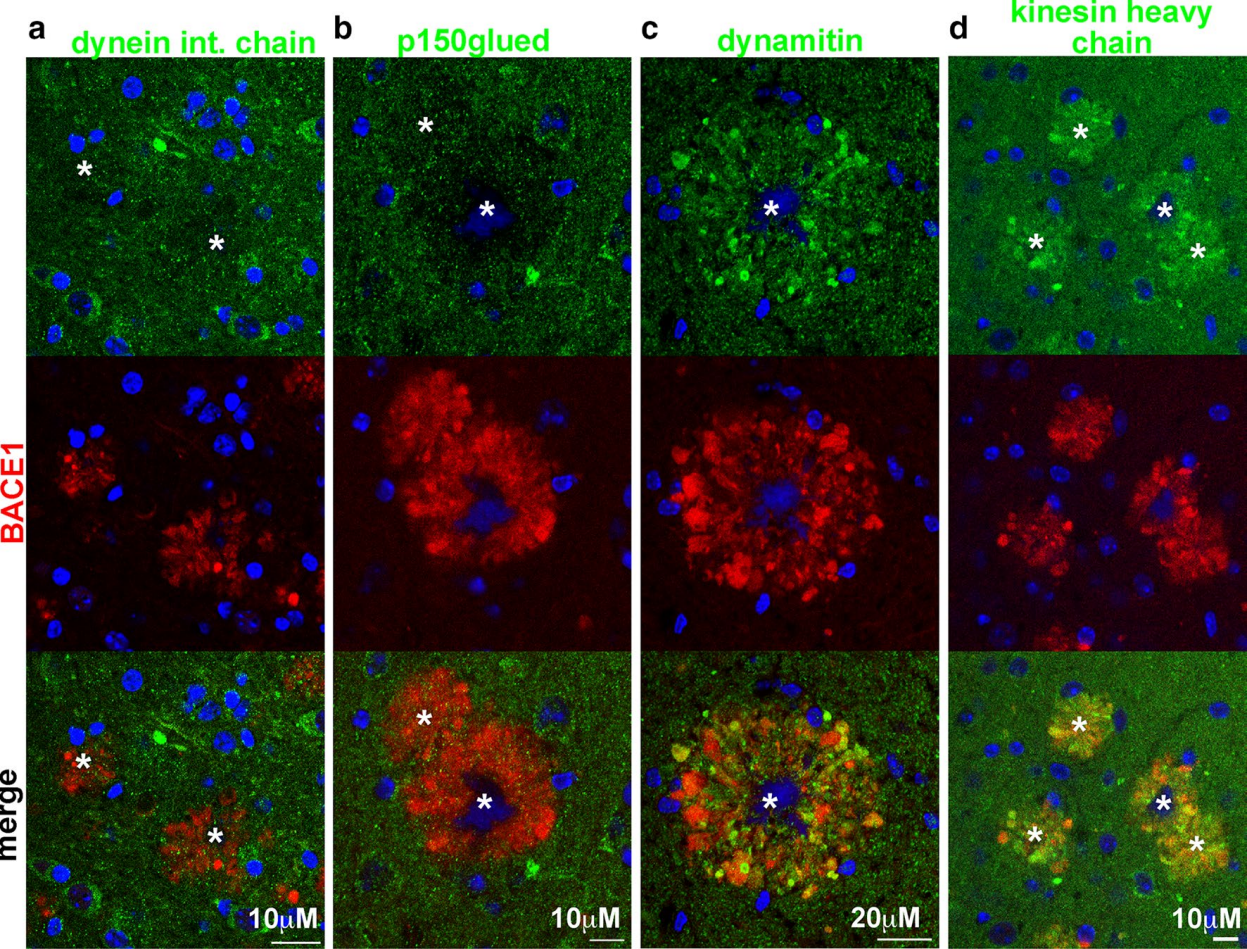
Microtubule motor proteins are aberrantly localized in BACE1-positive peri-plaque dystrophic neurites. Sections from 5XFAD mouse brains were stained with antibodies against BACE1 (*red*) and various components of the dynein–dynactin retrograde transport complex, dynein intermediate chain (*green*, **a**), p150glued (*green*, **b**), dynamitin (*green*, **c**), or the anterograde motor protein kinesin heavy chain (*green*, d). Representative amyloid deposits are shown; additional examples in Figs. S3 and S4. Dynein intermediate chain and p150glued were reduced in peri-plaque BACE1-positive dystrophic neurites, while dynamitin and kinesin heavy chain tended to accumulate in dystrophies and showed partial but limited co-localization with BACE1. These results together with evidence of microtubule disruption strongly suggest that axonal transport is impaired in dystrophic regions of peri-plaque axons. DAPI staining (*blue*) identified nuclei and autofluorescence of amyloid plaque cores (marked by *asterisks*). *Scale bars* 10 μm (**a**, **b**, **d**) and 20 μm (**c**)

Recent work has linked mutations in various proteins of the dynein–dynactin complex required for retrograde axonal transport to neurodegenerative disease in humans and mouse models [43]. If microtubule networks are disrupted in dystrophies around plaques, we reasoned that the microtubule motor proteins that move along them carrying cargo from the terminals back to the cell body could also be mis-localized, compounding the impaired removal of vesicles from dystrophic axons and terminals. This could lead to decreased turnover of proteins that are preferentially localized to presynaptic terminals, like BACE1 [25, 86]. To determine the localization pattern of microtubule motor proteins in peri-plaque dystrophies, we performed immunofluorescence microscopy of coronal brain sections from five individual 5- to 6-month-old 5XFAD mice using antibodies directed against cytoplasmic dynein intermediate chain (DIC), two subunits of dynactin (p150glued and dynamitin), and kinesin heavy chain (Figs. 5, S3, S4). DIC is a component of the retrograde microtubule motor, and while we observed some DIC staining in BACE1-positive dystrophic neurites, it was quite low compared to the amount of potential cargos such as BACE1 that accumulate in dystrophies (Figs. 5a, S3, S4). p150glued is required to initiate retrograde transport from the axon terminal [32, 37], stabilize microtubules in axons [30], and improve processivity of dynein [27, 35]. Importantly, p150glued was diminished in BACE1-positive peri-plaque dystrophies compared to normal surrounding neuropil (Figs. 5b, S3, S4), suggesting a deficiency of functional and active retrograde dynein–dynactin complexes in these dystrophies. In contrast, dynamitin staining was noticeably increased in some, but not all, BACE1-positive peri-plaque dystrophies (Figs. 5c, S3, S4). Dynamitin tethers the cargo-binding domain of dynactin to the microtubule and dynein-binding domain through its interactions with Arp1 and p150glued, respectively [5]. The increased dynamitin in peri-plaque dystrophies suggested that it may be trapped in either free form or in complexes with adaptor proteins and cargo, but lacking p150glued, dynamitin cannot be attached to dynein and transported with cargo back toward the soma. Disruption of microtubule networks in peri-plaque dystrophic neurites would also be predicted to have an effect on anterograde axonal transport by kinesins and cause kinesin mis-localization. Our immunofluorescence microscopy analysis showed that, similar to dynamitin, kinesin heavy chain accumulated in dystrophies surrounding plaques (Figs. 5d, S3, S4), supporting our hypothesis that peri-plaque presynaptic dystrophic neurites are deficient in microtubules and exhibit mis-localized microtubule motor proteins, conditions that would impair axonal transport.

### BACE1-positive peri-plaque dystrophies are presynaptic, but lack components of functional synapses

**Fig. 6:**
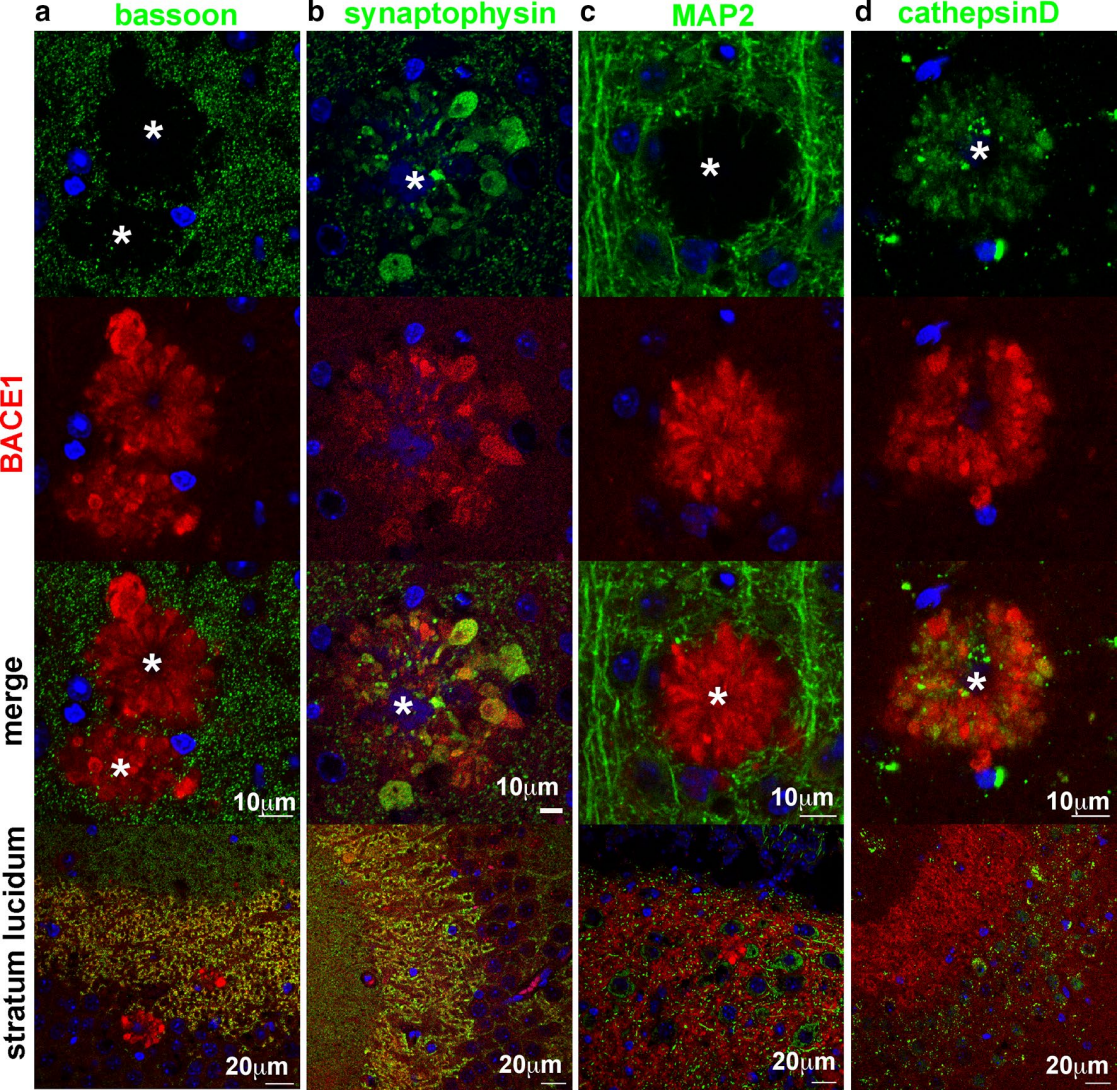
BACE1-positive peri-plaque dystrophies accumulate the presynaptic protein synaptophysin and the lysosomal protease cathepsin D, but lack the active zone protein bassoon. Sections from 5XFAD mouse brains were stained with antibodies against BACE1 (*red*) and active zone protein bassoon (*green*, **a**), presynaptic protein synaptophysin (*green*, **b**), somatodendritic marker MAP2, (*green*, **c**), or lysosomal protease cathepsin D (*green*, **d**). Representative amyloid deposits are shown; additional examples in Figs. S3 and S4. Both bassoon and synaptophysin co-localized extensively with BACE1 in presynaptic terminals in the stratum lucidum (*bottom row*), but bassoon and synaptophysin were absent and enriched in peri-plaque dystrophies, respectively (*top row*). The somatodendritic protein MAP2, which did not co-localize with BACE1 in the stratum lucidum (*bottom row*), was absent in peri-plaque dystrophies (*top row*). The lysosomal protease cathepsin D was found in neuronal soma as expected and did not co-localize with BACE1 in stratum lucidum (*bottom row*). However, cathepsin D tended to accumulate in some dystrophies, indicating aberrant localization of lysosomes in peri-plaque dystrophic neurites. These results suggest that BACE1-positive peri-plaque dystrophies are largely presynaptic axons and terminals, although dystrophic terminals are unlikely to have active synapses as they lack bassoon. DAPI staining (*blue*) identified nuclei and autofluorescence of amyloid plaque cores (marked by *asterisks*). *Scale bars* 10 μm in all frames except for the *bottom row*, which is 20 μm

To further characterize BACE1-positive peri-plaque dystrophic neurites in 5XFAD brain sections, we performed immunofluorescence microscopy with antibodies directed against BACE1 and several other neuronal proteins (Figs. 6, S3, S4), confirming that accumulation in dystrophies is specific to certain proteins but not others. Synaptophysin and bassoon, both presynaptic proteins, show strong co-localization with BACE1 in the stratum lucidum, the axon terminals of the mossy fiber pathway in the hippocampus (Fig. 6a, b; bottom row), confirming our previous results that BACE1 is normally localized in the presynaptic terminal [25]. As we have reported before [25, 86], and was recently confirmed [15, 50], many peri-plaque dystrophies appear to be axonal in origin, showing accumulations of the presynaptic protein synaptophysin (Fig. 6b), and a conspicuous absence of the somatodendritic marker MAP2 (Fig. 6c), which does not co-localize with BACE1 in the stratum lucidum (Fig. 6c; bottom panel). Surprisingly, given its strong co-staining with BACE1 in normal tissue of the stratum lucidum, bassoon appears to be entirely absent from peri-plaque dystrophies (Fig. 6a). Since bassoon is a well-established marker of synaptic active zones (reviewed in [6]), its absence from peri-plaque dystrophies indicates a lack of functional synapses in these regions. The toxic effect of amyloid plaques on synapses seems to be quite local, as the immunofluorescence signal for bassoon outside the immediate peri-plaque halo of the dystrophies appears as a normal punctate pattern. This is supported by other APP transgenic mouse studies showing that PSD95 was reduced by 60 % in the halo of oligomeric Aβ42 surrounding plaques, while 50 μm from the plaque it was found at normal levels [29].

### Immature pro-cathepsin D accumulates in 5XFAD mouse brain

**Fig. 7:**
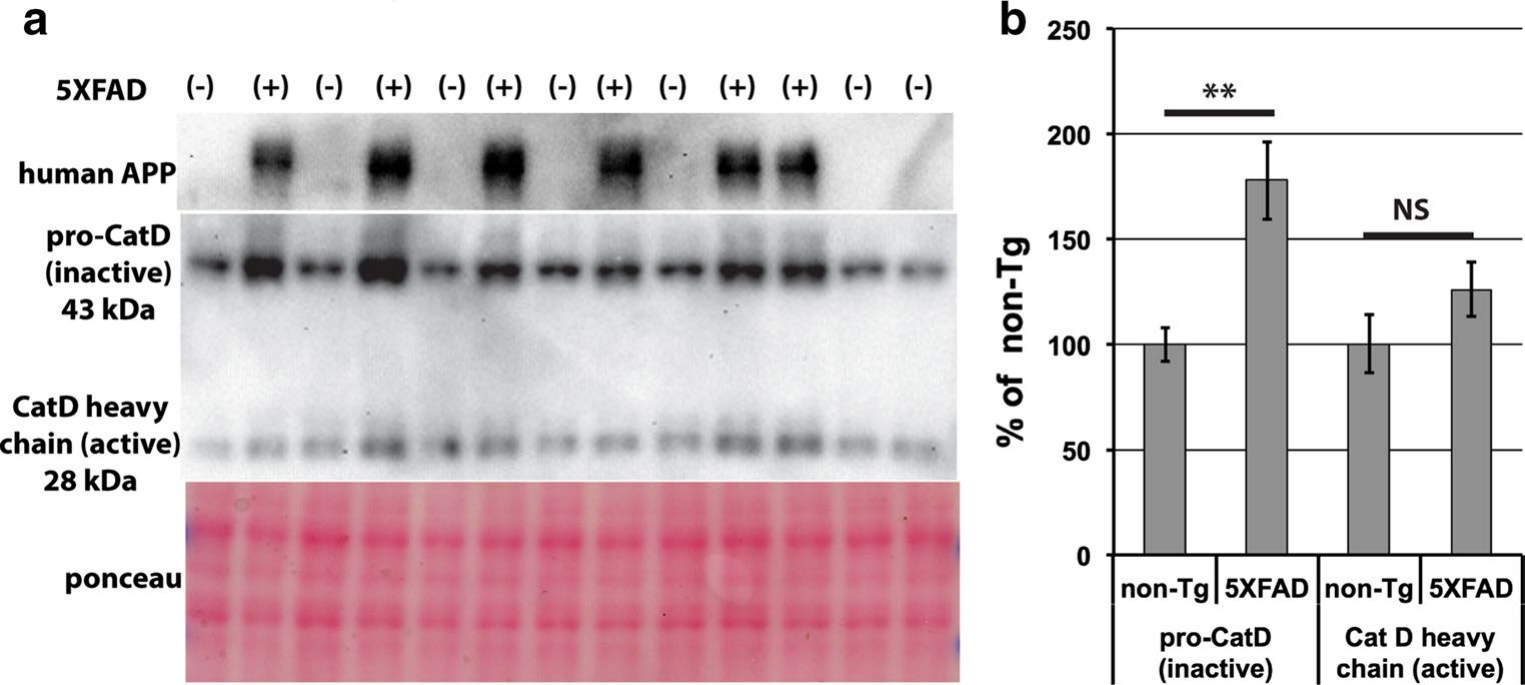
Levels of immature pro-cathepsin D are increased in 5XFAD mouse brain. **a** Cortices from 6-month 5XFAD (+) or non-transgenic (−) mice were homogenized and subjected to immunoblot analysis using antibodies against human APP (*top panel*; antibody 6E10) and the C-terminus of the lysosomal protease cathepsin D (CatD), which recognizes both immature (inactive) pro-CatD and mature (active) CatD heavy chain (*middle panel*). Ponceau S staining of the immunoblot was used to control for protein loading (*bottom panel*). Note that 43-kDa immature pro-CatD bands are more intense than 28 kDa mature CatD heavy chain bands, especially in 5XFAD cortices. **b** Band intensities from the immunoblot in **a** were quantified and normalized to total protein as determined by ponceau S staining intensity in respective lanes and then displayed as percentage of non-transgenic (non-Tg) control. Note that 5XFAD cortex levels of pro-CatD were nearly twofold higher than those in non-Tg cortex, while levels of CatD heavy chain were not significantly changed, suggesting that lysosomal maturation is impaired in 5XFAD brains. *Error bars* SEM; **p < 0.01; *NS* not significant

Cathepsin D is found in lysosomes, which normally localize to a peri-nuclear region of the soma rather than axon terminals, and cathepsin D does not co-localize with BACE1 in normal-appearing regions of the 5XFAD stratum lucidum (Fig. 6d, bottom panel). Although cathepsin D appeared somewhat elevated in 5XFAD BACE1-positive peri-plaque dystrophies, the patterns of cathepsin D and BACE1 localization did not overlap as extensively as those of synaptophysin and BACE1 (Figs. 6d, S3, S4). BACE1 is normally degraded through lysosomal pathways [26, 60], and thus presynaptic BACE1 is dependent on retrograde transport to the cell body for turnover. Early lysosomes, identified by Lamp1 expression, are found in dystrophic neurites [15, 25] but are deficient in cathepsins [15]. These studies together with our results suggest that microtubule disruption and motor protein mis-localization impair retrograde transport and hence lysosomal maturation, ultimately resulting in reduced degradation of BACE1 and other proteins in peri-plaque dystrophic neurites. The cathepsin D antibody that we used for our immunolocalization analysis (Fig. 6d) recognizes the C-terminus of both 43 kDa pro-cathepsin D and 28 kDa cathepsin D heavy chain, precluding our ability to determine whether peri-plaque cathepsin D was mature (active) or immature (inactive). To distinguish and quantify mature and immature forms of cathepsin D, we performed immunoblot analysis of brain homogenates from 6-month-old 5XFAD and non-transgenic mice using the cathepsin D antibody (Fig. 7a). We observed that 5XFAD brains had significantly increased levels of immature pro-cathepsin D compared to brains of non-transgenic littermates, while 5XFAD levels of mature cathepsin D heavy chain were unchanged (Fig. 7b). This result is consistent with the notion of impaired maturation of lysosomes in the brains of 5XFAD mice, which might contribute to accumulation of BACE1 and other proteins in peri-plaque dystrophic neurites.

### BACE1-positive peri-plaque dystrophic neurites are sources of increased production of BACE1-cleaved APP fragments and Aβ42

**Fig. 8:**
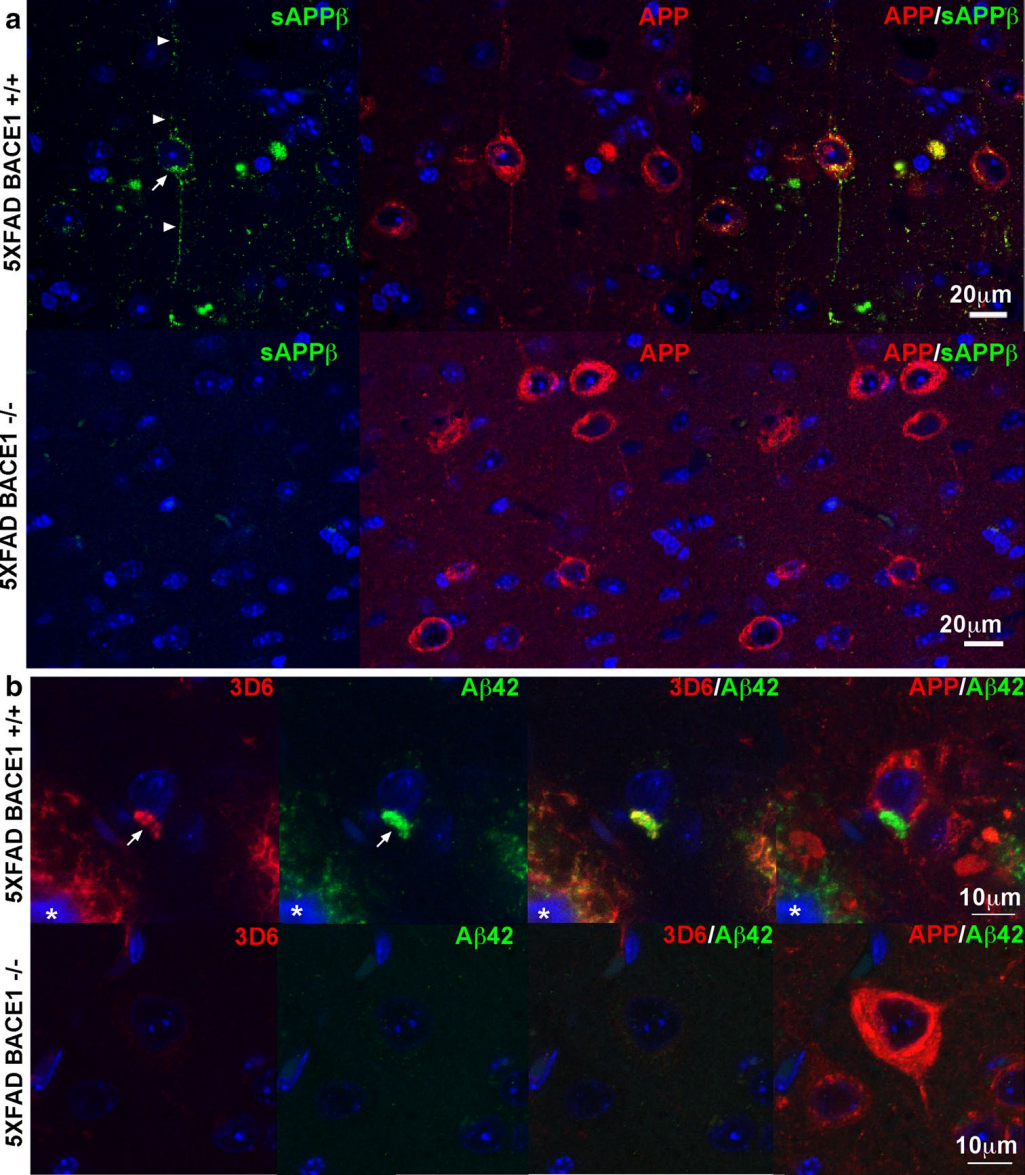
Neoepitope antibodies recognize BACE1-cleaved APP and Aβ but not full-length APP in vivo. Sagittal brain sections of 5XFAD;BACE1+/+ and 5XFAD;BACE1−/− mice were immunostained with neoepitope antibodies to **a** the free C-terminus of BACE1-cleaved APP ectodomain ending in the Swedish mutation, sAPPβ (antibody ANJJ [45, 46], *green*), **b** the free N-terminus of BACE1-cleaved Aβ (antibody 3D6 [23], *red*), or **c** the free C-terminus of γ-secretase-cleaved Aβ42 (*green*). Sections were also co-immunostained with an antibody recognizing full-length APP (antibody Karen [64], *red*), then imaged by confocal microscopy. All three neoepitope immunoreactivities were present in soma (*arrows*), and processes in the case of sAPPβ (*arrowheads*), of pyramidal neurons in the cortex of 5XFAD;BACE1+/+ mice, while none were detected in 5XFAD;BACE1−/− cortex, despite overexpression of transgenic APP in pyramidal neurons of both genotypes. These data confirm that ANJJ, 3D6, and Aβ42 antibodies are selective for their respective neoepitopes and do not cross-react to full-length APP. DAPI staining (*blue*) identified nuclei and autofluorescence of amyloid deposit cores (marked by *asterisks*). Note that amyloid deposits are completely lacking in 5XFAD;BACE1−/− cortex, as previously reported [41]. *Scale bars* 10 μm (**b**), 20 μm (**a**)

Our observations of BACE1 accumulation around plaques led us to the hypothesis that elevated levels of BACE1, APP [25, 86], and PS1 [81] found in peri-plaque dystrophic neurites would cause increased BACE1 processing of APP and Aβ production, thus exacerbating AD pathogenesis. However, direct evidence that elevated BACE1 cleaves APP and leads to increased Aβ formation in dystrophic neurites has been lacking. To address this question, we made use of several antibodies specific to neoepitopes of APP-derived fragments revealed by BACE1 and γ-secretase processing. BACE1 cleavage of APP generates two products, the N-terminal soluble APP ectodomain fragment, sAPPβ, and the membrane bound C-terminal fragment, C99. The antibody ANJJ [45] recognizes the free C-terminus of sAPPβ created by BACE1 processing of APP with the Swedish familial AD mutation [38], which is expressed in 5XFAD mice [40]. To validate ANJJ for tissue immunostaining and verify that it does not recognize unprocessed full-length APP, we co-stained sagittal brain sections from 9-month-old 5XFAD;BACE1+/+ and 5XFAD;BACE1−/− bigenic mice with ANJJ and an antibody to an N-terminal epitope of full-length APP [64] and performed immunofluorescence confocal microscopy (Fig. 8). We observed punctate sAPPβ immunoreactivity of ANJJ in the soma and processes of 5XFAD;BACE1+/+ large pyramidal neurons in the cortex (Figs. 8a, upper panels; S4), the primary cells that express the 5XFAD transgenes in the brain [40]. The punctate intracellular immunostaining pattern for sAPPβ was expected, since BACE1 cleaves APP in endosomes [45, 66] to create the sAPPβ neoepitope and release the soluble APP ectodomain into the endosome lumen for eventual secretion into the extracellular milieu. The immunoreactivity of full-length APP exhibited a wider spatial distribution in the neuron compared to that of sAPPβ, indicating cell surface localization in addition to intracellular labeling. In contrast, sAPPβ immunosignal was completely absent in 5XFAD;BACE1−/− brain sections (Fig. 8a, lower panels), indicating the absence of the sAPPβ free C-terminal neoepitope as expected for BACE1-deficient mice [34], even though full-length APP immunoreactivity throughout the neuron was similar to that observed in 5XFAD;BACE1+/+ sections. These results demonstrate conclusively that the ANJJ antibody only recognizes the BACE1-cleaved sAPPβ C-terminal neoepitope and does not cross-react to full-length APP in brain sections.

We also performed immunofluorescence microscopy on 5XFAD;BACE1+/+ and 5XFAD;BACE1−/− brain sections incubated with antibodies recognizing neoepitopes on the free N-terminus of BACE1-cleaved Aβ (3D6) [23] and the free C-terminus of γ-secretase-cleaved Aβ ending in amino acid 42 (Aβ42), co-stained with the full-length APP antibody (Fig. 8b). As with sAPPβ immunosignal, we observed intraneuronal 3D6 and Aβ42 neoepitope immunostaining only in large pyramidal cortical neurons of 5XFAD;BACE1+/+ mice, while those of 5XFAD;BACE1−/− mice were completely devoid of either 3D6 or Aβ42 neoepitope immunoreactivities (Fig. 8b) even though they showed robust full-length APP labeling. Taken together, these results fully validate the 3D6 and Aβ42 neoepitope antibodies for tissue immunostaining and confirm that they do not label full-length APP.

**Fig. 9:**
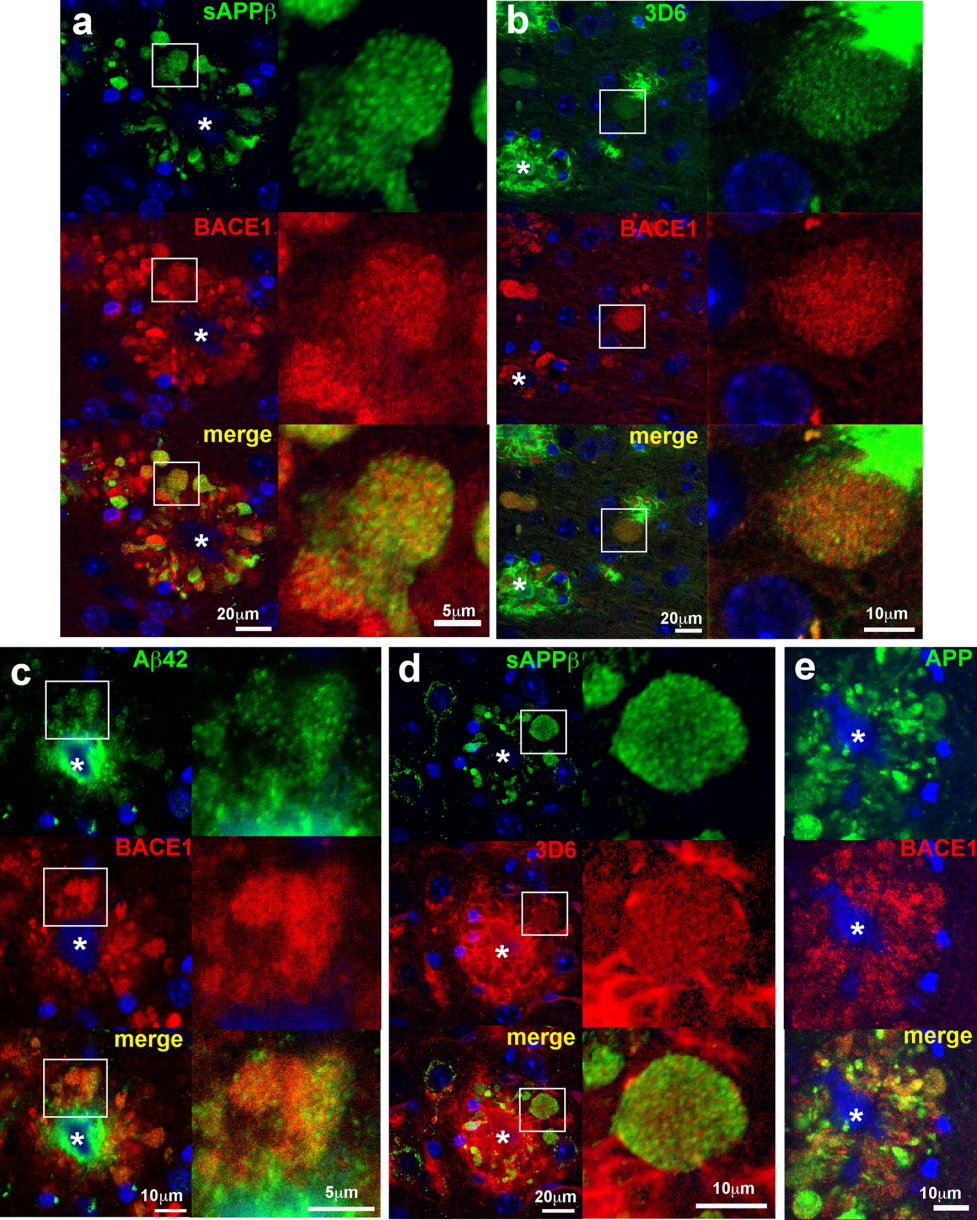
BACE1 accumulation in peri-plaque dystrophic neurites increases BACE1 cleavage of APP and generation of Aβ42. Sagittal brain sections of 5XFAD mice were co-immunostained with antibodies recognizing BACE1 and cleaved APP fragment neoepitopes (sAPPβ, 3D6, Aβ42) validated in Fig. 8. **a** The BACE1-cleaved free C-terminal neoepitope of sAPPβ (ANJJ, *green*) showed robust co-localization with elevated BACE1 (*red*) in peri-plaque dystrophic neurites. Note the intense sAPPβ immunoreactivity in dystrophies, while little if any sAPPβ signal is present in surrounding neuropil, demonstrating dramatically elevated BACE1 cleavage of APP in BACE1-positive dystrophic neurites near amyloid deposits. **b** Antibody 3D6 (*green*), which recognizes the free N-terminal neoepitope of BACE1-cleaved Aβ, also co-localized with elevated BACE1 (*red*) in dystrophic neurites, confirming increased BACE1 activity and Aβ generation in dystrophies. Antibody 3D6 also detects amyloid plaques, as expected (example marked with *asterisk*). **c** Like 3D6, an antibody recognizing the free C-terminal neoepitope generated after γ-secretase cleavage of Aβ42 (*green*) co-localized with elevated BACE1 (*red*) in peri-plaque dystrophies, and detected amyloid deposits, as expected (*asterisk*). **d** Co-immunostaining also demonstrated that both sAPPβ (*green*) and 3D6 (Aβ, *red*) neoepitopes co-localized in peri-plaque dystrophies. **e** Co-immunostaining with antibodies against BACE1 (*red*) and APP (Karen, *green*) also showed a high level of co-localization. Note that all cleaved APP fragment neoepitopes and BACE1 show a punctate immunostaining pattern in dystrophies, suggesting that they are localized to vesicles such as endosomes, while APP immunostaining appears more uniform, implying greater cell surface localization. *Panels* in the *right column* of each set are high-magnification images of the *boxed regions* in the frames of the *left column*. DAPI staining (*blue*) identified nuclei and autofluorescence of amyloid deposit cores (marked by *asterisks*). *Scale bar* sizes are indicated in *bottom panels* for images in *each column*

Next, using our validated neoepitope antibodies we sought to determine whether elevated BACE1 in peri-plaque dystrophies was associated with increased BACE1 processing of APP and Aβ generation. Importantly, sagittal brain sections from 5- to 6-month and 9-month-old 5XFAD mice co-incubated with sAPPβ neoepitope and BACE1 antibodies showed robust punctate sAPPβ immunoreactivity, likely representing endosomal localization, within peri-plaque dystrophies that significantly co-localized with BACE1 immunosignal (Figs. 9a, S4). sAPPβ immunoreactivity was very intense in peri-plaque dystrophies, compared to the very low sAPPβ immunosignal in the surrounding neuropil, indicating that BACE1 processing of APP was dramatically increased in the BACE1-positive dystrophic neurites near amyloid deposits. We noted that peri-plaque dystrophies exhibited variable levels of transgenic sAPPβ immunoreactivity, presumably because the Thy1 transgene promoter is expressed in some, but not all, cortical neurons [36]. Similar to the sAPPβ immunostaining pattern, we observed increased punctate 3D6 and Aβ42 neoepitope immunosignals that co-localized significantly with BACE1 immunoreactivity in peri-plaque dystrophies (Figs. 9b, c, respectively; S4), suggesting that BACE1 accumulation elevated Aβ production. Although 3D6 and Aβ42 immunosignal intensities in dystrophies were weaker than those in amyloid deposits, they were obviously elevated compared to surrounding neuropil, especially at higher magnifications (right panels, 9b, c). Additionally, sAPPβ and 3D6 neoepitope immunoreactivities co-localized significantly in peri-plaque dystrophies (Fig. 9d). Co-immunostaining of 5XFAD brain sections with full-length APP and BACE1 antibodies confirmed that APP and BACE1 accumulate together in peri-plaque dystrophies (Fig. 9e), implicating both in plaque progression.

Taken together, our results suggest that elevated BACE1 in dystrophic neurites around plaques is associated with increased BACE1 cleavage of APP and Aβ generation. The presence of neoepitope N-terminal and C-terminal APP processing products in peri-plaque dystrophies strongly supports the hypothesis that BACE1 and γ-secretase are active in dystrophies, although we cannot formally exclude the possibility that APP processing occurs elsewhere in the cell followed by APP product trafficking and accumulation in dystrophies. Additionally, our results show that peri-plaque dystrophic neurites accumulate Aβ42, which if released into the extracellular space could contribute to plaque growth and further neuritic dystrophy.

## Discussion

In this study, we provide evidence that Aβ mediates microtubule disruption and microtubule-based transport impairment leading to dystrophic neurite formation, BACE1 accumulation, increased BACE1 cleavage of APP and Aβ production. Primary neurons treated with Aβ42 oligomers exhibited disrupted microtubules, neuritic beading, and reduced BACE1-YFP trafficking after only a short Aβ42 exposure in vitro. In the 5XFAD mouse model of amyloid pathology, presynaptic dystrophic neurites surrounding plaques showed BACE1 accumulation that correlated with aberrant localization patterns of tubulins, microtubule motor proteins, and synaptic and cell body proteins. Tubulin was largely absent from human AD and 5XFAD BACE1-positive peri-plaque dystrophies and showed increased BACE1:tubulin ratios and inverse relationships between BACE1 and tubulin. Although 5XFAD peri-plaque dystrophic neurites accumulated the lysosomal protease cathepsin D, it largely appeared to be immature pro-cathepsin D, suggesting that impaired lysosomal maturation correlates with BACE1 accumulation. By EM, peri-plaque dystrophic axons lacked intact microtubules, consistent with reduced tubulin by immunofluorescence microscopy. Remarkably, the toxic effects of amyloid on dystrophies were highly localized to the immediate vicinity of the plaque, with neighboring axons and terminals appearing normal. Multi-photon confocal microscopy 3D-reconstructions of live 5XFAD;BACE1-YFP brain slices invariably showed physical contact between BACE1-YFP-positive dystrophies and amyloid deposits. Most importantly, peri-plaque dystrophies displayed BACE1 and APP accumulation, increased BACE1 cleavage of APP, and elevated Aβ42 generation.

Taken together, our results suggest the following working hypothesis of presynaptic dystrophic neurite formation and plaque progression (Fig. S6). During early stages of amyloid deposition, a plaque nidus forms stochastically in the parenchyma and is too small to be significantly toxic to axons and terminals. Following further Aβ addition, the plaque grows large enough to come into close proximity to a nearby axon or terminal and causes neurotoxicity derived from high local concentrations of either soluble Aβ oligomers or insoluble Aβ fibrils. An as yet undefined cascade is triggered that leads to local destabilization and/or depolymerization of microtubules in the axon; microtubules in more distant regions of the axon are unaffected. Microtubule-dependent axonal transport is impaired locally in the region of the axon nearest the plaque in which microtubules are disrupted, although transport is normal in segments further away from the plaque. Vesicles proximal and distal to the plaque undergoing anterograde and retrograde transport, respectively, detach from the ends of disrupted microtubules and begin to accumulate causing swelling and axonal dystrophy nearest the plaque. The accumulation of vesicles containing APP, BACE1, and γ-secretase leads to increased APP processing and Aβ generation in the dystrophic region of the axon and may accelerate growth and development of the nearby plaque. This in turn results in a feed-forward mechanism of increased axonal dystrophy, BACE1 and APP accumulation, and Aβ generation. Together with dysfunction associated with axonal swelling and impaired transport, this cascade is likely to exacerbate downstream neurodegeneration leading to cognitive deficits. Despite being likely of presynaptic origin, peri-plaque dystrophies lack a structural protein of active zones, bassoon, suggesting that they are not functional in synaptic transmission. Importantly, this cascade would disrupt vesicular trafficking from the soma to the terminal and vice versa. Protein turnover could be inhibited resulting in accumulation of certain proteins like BACE1, and loss of others according to the specific cellular mechanisms by which their transport and degradation are controlled. In future work, it will be important to determine the potential roles of other major players in dystrophic neurite generation, such as Reticulon 3, which has been implicated in dystrophic neurite formation [19, 52, 53], or the recently discovered η-secretase processing of APP [73] (see Supplementary Text, Fig. S7), in the processes that we describe here.

Our data suggest that BACE1-positive peri-plaque dystrophic neurites are axonal or terminal in origin due to the presence of the predominantly axonal protein neurofilament NF-M [25, 74], synaptic vesicle protein synaptophysin (Fig. 6b) [25, 85, 86] and myelination (Fig. 4), and absence of the somatodendritic protein MAP2 (Fig. 6c) [25, 85, 86]. Moreover, endogenous BACE1 is highly concentrated in vesicles of presynaptic terminals [25]. Consistent with our results, other APP transgenic mice have peri-plaque APP-positive dystrophies that stain for synaptophysin and the presynaptic vesicular glutamate transporter VGLUT1 but lack MAP2, and also have dystrophies that are myelinated and accumulate autophagic intermediates by EM [50]. VGLUT1 and other presynaptic markers including growth-associated protein 43 (GAP43), glutamic acid decarboxylase 67 (GAD67), and choline acetyltransferase (ChAT) have also been shown to co-localize with BACE1 in dystrophic neurites, supporting their presynaptic/axonal origin [85]. Although lack of BACE1 and MAP2 co-localization does not guarantee that BACE1-positive dystrophies are not dendritic, the robust co-localization of BACE1 with five presynaptic markers strongly suggests presynaptic origin. The absence of synaptic active zone scaffold protein bassoon in BACE1-positive dystrophies (Fig. 6a) could be caused by loss of normal synaptic structure. The post-synaptic density protein PSD95 is also reduced around plaques [29], supporting this hypothesis. Thus, the majority of evidence suggests that BACE1-positive peri-plaque dystrophies are presynaptic in origin, although we cannot exclude the possibility that some BACE1-positive dystrophic neurites are post-synaptic/dendritic in nature.

There has been much debate about whether plaques are a toxic agent in Alzheimer’s disease, or a mechanism for the brain to sequester amyloid in a less harmful form. Early reports indicated that plaque load did not correlate well with cognitive impairment [1, 59]. However, recent work has shown that cerebral amyloid deposition identified by amyloid-PET imaging in cognitively normal and mildly impaired individuals predicts conversion to AD [22]. Furthermore, numerous studies have documented toxic effects such as mitochondrial loss [77], oxidative damage [78], synapse loss [29], and spine loss [75] in the immediate vicinity of plaques. In our study, we found that dystrophic neurites appear to be in contact with amyloid plaques at the ultrastructural level (Fig. 4), by immunofluorescence microscopy (Figs. 3, 5, 6, 9, S3, S4) and in live slices by multi-photon confocal microscopy (Fig. S5; Videos S1, S2), whereas areas of neuropil slightly further away are morphologically normal. Interestingly, a study using an antibody specific to oligomeric Aβ (NAB61) determined that a halo of oligomeric Aβ extends ~6.5 μm beyond the edge of the fibrillar Aβ core [29]. These results suggest that plaques are sources of soluble Aβ oligomers, widely thought to be the toxic species in AD, that are in dynamic equilibrium with insoluble Aβ deposits, thus creating high local concentrations of neurotoxic Aβ.

Previous studies have reported that soluble Aβ levels in the brain are in the picomolar range [33, 76]. However, this estimate is based on biochemical isolation of Aβ from post-mortem brain lysates and thus is the *average* concentration of soluble Aβ in the brain, but it may not reflect the *local* soluble Aβ concentration near the plaque. To our knowledge, *absolute soluble Aβ concentrations in the immediate vicinity of the plaque have not been quantified*. Although a halo of Aβ oligomers emanating from individual plaques has been demonstrated [29], the *absolute* quantification of soluble peri-plaque Aβ concentrations was not performed in this study. Thus, we suggest that *local* peri-plaque concentrations of soluble Aβ may be much higher than *average* Aβ concentrations in the brain. If so, high *local* soluble Aβ concentrations may cause microtubule disruption and neuritic swelling in the portion of the neurite that is very near the plaque, but parts of the neurite that are farther away would be exposed to lower soluble Aβ concentrations that may not induce neuritic dystrophy. Such a localized effect on only the peri-plaque portion of the neurite may not induce cell death processes in the distant cell body, at least not initially. This scenario is consistent with both our in vitro and in vivo results.

Although it is challenging to measure Aβ oligomer concentrations in the halo surrounding the amyloid deposit, the local concentrations of Aβ may reach very high values approaching the plaque. We used relatively high concentrations of Aβ42 oligomers (1–10 μM) in our primary neuron experiments to model the high local oligomeric Aβ42 concentration in the immediate vicinity of the amyloid deposit. It is unlikely that microtubule disruption (Figs. 1, 2) and axonal transport impairment (Fig. S2) in Aβ42-treated primary neurons were the result of cell death processes, since propidium iodide staining showed no increase in dead cells in the Aβ42-treated cultures (Fig. S1). Additionally, we previously showed that 5 days of treatment with 1 or 2 μM Aβ42 oligomers caused no significant increase of activated caspase 3 in primary neurons [49]. Microtubule disruption may occur at concentrations lower than 1 μM Aβ42, although we have not tested this yet. However, a previous study has shown that 0.5 μM Aβ42 oligomers does not appear to disrupt microtubules in primary neurons [7], suggesting that 1 μM Aβ42 may be a threshold concentration for microtubule disruption. The relationship between our in vitro and in vivo results still requires further investigation, but even in aged 5XFAD mice with high levels of soluble Aβ42, we observe microtubule disruption and aberrant tubulin localization only in the immediate vicinity of amyloid deposits, suggesting that high Aβ42 concentrations are necessary for these effects. When conditional expression of APP in transgenic mouse brain is reduced by over 90 % with doxycycline treatment, Aβ40 and Aβ42 levels in interstitial fluid drop by about 70 %, suggesting that while plaques are fairly stable over time, they do contribute to soluble Aβ in the brain [13]. The hypothesis has been advanced that the toxic species of Aβ is generated in plaques, as the Aβ bound there over time becomes modified, gaining neurotoxicity [83]. Alternatively, insoluble fibrillar Aβ in plaques could be directly neurotoxic, or indirectly induce toxicity through secondary mechanisms such as neuroinflammation. Much future research remains to precisely define the exact neurotoxic species of Aβ in AD.

The cellular and molecular mechanisms leading to amyloid-induced microtubule disruption and axonal dystrophy are not yet clear. Recent studies investigating the effects of Aβ42 oligomers on Tau mis-sorting indicate that the microtubule-severing enzyme spastin mediates microtubule breakdown in dendrites [82]; however, a role for this mechanism in axons is unclear. It is unlikely that Tau is directly involved in the generation of dystrophic neurites, as hAPP; Tau−/− mice have the same plaque area and same percentage of plaques with dystrophic neurites as hAPP; Tau+/+ mice despite having improved survival and cognitive function [47]. Importantly, the absence of Tau did not affect the generation of APP-positive dystrophies that, based upon our results, were also likely BACE1-positive (Fig. 9e). These results suggest that Tau has an important role in Aβ-related cognitive deficits, but Tau does not appear central to the effects of amyloid deposits on dystrophic neurite formation. It is more likely that depolymerization of microtubules affects Tau phosphorylation, mis-sorting, and aggregation.

Aβ42 oligomers have been proposed to mediate toxicity through a number of extracellular receptors such as glutamate receptors, p75 neurotrophin receptors, nicotinic acetylcholine receptors, amylin receptors, the receptor for advanced glycation end products (RAGE), insulin and IGF receptors, and others (reviewed in [42]). Activation of these receptors leads to changes in LTP, modulation of various signaling cascades, including Jun kinase, p38, MAPKs and p53, as well as caspase activation (reviewed in [42]), some of which can cause apoptosis and cell death. Oligomeric Aβ42 leads to synaptic dysfunction that correlates with cognitive decline, perhaps mediated through effects on NMDA receptors [63]. One study showed that NMDA receptor antagonists and GSK3β inhibitors prevented Aβ oligomer-induced disruption of axonal trafficking, suggesting that these pathways are involved [7].

The identity of BACE1-positive vesicles in peri-plaque dystrophic axons is not yet clear. Our previous EM analysis indicates that the dystrophies are heterogeneous, some containing smaller, clear vesicles, possibly endosomes, and others containing larger, multi-lamellar, electron-dense vesicles that may be autophagic/lysosomal intermediates [25]. By immuno-EM, BACE1 is predominantly found in dystrophies with small, electron-translucent vesicles. Recent work indicates that large numbers of Lamp1-positive immature lysosomes accumulate around plaques, and that BACE1 co-localizes with Lamp1, suggesting BACE1 accumulation in immature lysosomes [15]. Although we have observed Lamp1 [25] and cathepsin D (this study) accumulation in peri-plaque dystrophies, co-localization of these lysosomal proteins with BACE1 is limited, suggesting that BACE1 trafficking to lysosomes is impaired. Additionally, our data showing elevated immature proteolytically inactive pro-cathepsin D in 5XFAD brains support the hypothesis that lysosomal maturation is impaired, which could reduce the degradation of BACE1 and cause it to accumulate. Further studies are needed to better characterize the heterogeneity of peri-plaque dystrophies, and determine its causes and consequences.

While we clearly observed Aβ42 in presynaptic dystrophic neurites around plaques, future investigation must determine whether these dystrophies are a significant source of Aβ42 for plaque growth. Local Aβ generation should be higher in plaque-rich than in plaque-poor brain regions, and in older APP transgenic mice with elevated BACE1, compared to young animals. If so, these data could indicate that stabilization of microtubules might be beneficial in slowing pathology and plaque growth, and perhaps preserve neuronal and cognitive function for the treatment of AD.

## Electronic supplementary material

Below is the link to the electronic supplementary material.

Supplementary material 1 (PDF 367228 kb)

Supplementary material 2 (MOV 4462 kb)

Supplementary material 3 (MOV 4479 kb)

## References

[1] Arriagada PV, Growdon JH, Hedley-Whyte ET, Hyman BT (1992). Neurofibrillary tangles but not senile plaques parallel duration and severity of Alzheimer’s disease. Neurology.

[2] Blasko I, Beer R, Bigl M, Apelt J, Franz G, Rudzki D, Ransmayr G, Kampfl A, Schliebs R (2004). Experimental traumatic brain injury in rats stimulates the expression, production and activity of Alzheimer’s disease beta-secretase (BACE-1). J Neural Transm.

[3] Boissonneault V, Plante I, Rivest S, Provost P (2009). MicroRNA-298 and microRNA-328 regulate expression of mouse beta-amyloid precursor protein-converting enzyme 1. J Biol Chem.

[4] Cai H, Wang Y, McCarthy D, Wen H, Borchelt DR, Price DL, Wong PC (2001). BACE1 is the major beta-secretase for generation of Abeta peptides by neurons. Nat Neurosci.

[5] Cheong FK, Feng L, Sarkeshik A, Yates JR, Schroer TA (2014). Dynactin integrity depends upon direct binding of dynamitin to Arp1. Mol Biol Cell.

[6] Clarke GL, Chen J, Nishimune H (2012). Presynaptic active zone density during development and synaptic plasticity. Front Mol Neurosci.

[7] Decker H, Lo KY, Unger SM, Ferreira ST, Silverman MA (2010). Amyloid-beta peptide oligomers disrupt axonal transport through an NMDA receptor-dependent mechanism that is mediated by glycogen synthase kinase 3beta in primary cultured hippocampal neurons. J Neurosci.

[8] Di Fede G, Catania M, Morbin M, Rossi G, Suardi S, Mazzoleni G, Merlin M, Giovagnoli AR, Prioni S, Erbetta A, Falcone C, Gobbi M, Colombo L, Bastone A, Beeg M, Manzoni C, Francescucci B, Spagnoli A, Cantu L, Del Favero E, Levy E, Salmona M, Tagliavini F (2009). A recessive mutation in the APP gene with dominant-negative effect on amyloidogenesis. Science.

[9] Dickson DW (1997). The pathogenesis of senile plaques. J Neuropathol Exp Neurol.

[10] Emery DG, Lucas JH (1995). Ultrastructural damage and neuritic beading in cold-stressed spinal neurons with comparisons to NMDA and A23187 toxicity. Brain Res.

[11] Evans NA, Facci L, Owen DE, Soden PE, Burbidge SA, Prinjha RK, Richardson JC, Skaper SD (2008). Abeta(1-42) reduces synapse number and inhibits neurite outgrowth in primary cortical and hippocampal neurons: a quantitative analysis. J Neurosci Methods.

[12] Faghihi MA, Zhang M, Huang J, Modarresi F, Van der Brug MP, Nalls MA, Cookson MR, St-Laurent G, Wahlestedt C (2010). Evidence for natural antisense transcript-mediated inhibition of microRNA function. Genome Biol.

[13] Fowler SW, Chiang AC, Savjani RR, Larson ME, Sherman MA, Schuler DR, Cirrito JR, Lesne SE, Jankowsky JL (2014). Genetic modulation of soluble Abeta rescues cognitive and synaptic impairment in a mouse model of Alzheimer’s disease. J Neurosci.

[14] Fukumoto H, Cheung BS, Hyman BT, Irizarry MC (2002). Beta-secretase protein and activity are increased in the neocortex in Alzheimer disease. Arch Neurol.

[15] Gowrishankar S, Yuan P, Wu Y, Schrag M, Paradise S, Grutzendler J, De Camilli P, Ferguson SM (2015). Massive accumulation of luminal protease-deficient axonal lysosomes at Alzheimer’s disease amyloid plaques. Proc Natl Acad Sci USA.

[16] Haass C, Selkoe DJ (2007). Soluble protein oligomers in neurodegeneration: lessons from the Alzheimer’s amyloid beta-peptide. Nat Rev Mol Cell Biol.

[17] Hebert SS, Horre K, Nicolai L, Papadopoulou AS, Mandemakers W, Silahtaroglu AN, Kauppinen S, Delacourte A, De Strooper B (2008). Loss of microRNA cluster miR-29a/b-1 in sporadic Alzheimer’s disease correlates with increased BACE1/beta-secretase expression. Proc Natl Acad Sci USA.

[18] Holsinger RMD, McLean CA, Beyreuther K, Masters CL, Evin G (2002). Increased expression of the amyloid precursor beta-secretase in Alzheimer’s disease. Ann Neurol.

[19] Hu X, Shi Q, Zhou X, He W, Yi H, Yin X, Gearing M, Levey A, Yan R (2007). Transgenic mice overexpressing reticulon 3 develop neuritic abnormalities. EMBO J.

[20] Hussain I, Powell D, Howlett DR, Tew DG, Meek TD, Chapman C, Gloger IS, Murphy KE, Southan CD, Ryan DM, Smith TS, Simmons DL, Walsh FS, Dingwall C, Christie G (1999). Identification of a novel aspartic protease (Asp 2) as beta-secretase. Mol Cell Neurosci.

[21] Ikegami K, Kato S, Koike T (2004). *N*-alpha-*p*-tosyl-l-lysine chloromethyl ketone (TLCK) suppresses neuritic degeneration caused by different experimental paradigms including in vitro Wallerian degeneration. Brain Res.

[22] Jack CR, Barrio JR, Kepe V (2013). Cerebral amyloid PET imaging in Alzheimer’s disease. Acta Neuropathol.

[23] Johnson-Wood K, Lee M, Motter R, Hu K, Gordon G, Barbour R, Khan K, Gordon M, Tan H, Games D, Lieberburg I, Schenk D, Seubert P, McConlogue L (1997). Amyloid precursor protein processing and A beta42 deposition in a transgenic mouse model of Alzheimer disease. Proc Natl Acad Sci USA.

[24] Jonsson T, Atwal JK, Steinberg S, Snaedal J, Jonsson PV, Bjornsson S, Stefansson H, Sulem P, Gudbjartsson D, Maloney J, Hoyte K, Gustafson A, Liu Y, Lu Y, Bhangale T, Graham RR, Huttenlocher J, Bjornsdottir G, Andreassen OA, Jonsson EG, Palotie A, Behrens TW, Magnusson OT, Kong A, Thorsteinsdottir U, Watts RJ, Stefansson K (2012). A mutation in APP protects against Alzheimer’s disease and age-related cognitive decline. Nature.

[25] Kandalepas PC, Sadleir KR, Eimer WA, Zhao J, Nicholson DA, Vassar R (2013). The Alzheimer’s beta-secretase BACE1 localizes to normal presynaptic terminals and to dystrophic presynaptic terminals surrounding amyloid plaques. Acta Neuropathol.

[26] Kang EL, Cameron AN, Piazza F, Walker KR, Tesco G (2010). Ubiquitin regulates GGA3-mediated degradation of BACE1. J Biol Chem.

[27] Kardon JR, Reck-Peterson SL, Vale RD (2009). Regulation of the processivity and intracellular localization of *Saccharomyces cerevisiae* dynein by dynactin. Proc Natl Acad Sci USA.

[28] Kawataki T, Osafune K, Suzuki M, Koike T (2008). Neuronal maturation-associated resistance of neurite degeneration caused by trophic factor deprivation or microtubule-disrupting agents. Brain Res.

[29] Koffie RM, Meyer-Luehmann M, Hashimoto T, Adams KW, Mielke ML, Garcia-Alloza M, Micheva KD, Smith SJ, Kim ML, Lee VM, Hyman BT, Spires-Jones TL (2009). Oligomeric amyloid beta associates with postsynaptic densities and correlates with excitatory synapse loss near senile plaques. Proc Natl Acad Sci USA.

[30] Lazarus JE, Moughamian AJ, Tokito MK, Holzbaur EL (2013). Dynactin subunit p150(Glued) is a neuron-specific anti-catastrophe factor. PLoS Biol.

[31] Li R, Lindholm K, Yang LB, Yue X, Citron M, Yan R, Beach T, Sue L, Sabbagh M, Cai H, Wong P, Price D, Shen Y (2004). Amyloid beta peptide load is correlated with increased beta-secretase activity in sporadic Alzheimer’s disease patients. Proc Natl Acad Sci USA.

[32] Lloyd TE, Machamer J, O’Hara K, Kim JH, Collins SE, Wong MY, Sahin B, Imlach W, Yang Y, Levitan ES, McCabe BD, Kolodkin AL (2012). The p150(Glued) CAP-Gly domain regulates initiation of retrograde transport at synaptic termini. Neuron.

[33] Lue LF, Kuo YM, Roher AE, Brachova L, Shen Y, Sue L, Beach T, Kurth JH, Rydel RE, Rogers J (1999). Soluble amyloid beta peptide concentration as a predictor of synaptic change in Alzheimer’s disease. Am J Pathol.

[34] Luo Y, Bolon B, Kahn S, Bennett BD, Babu-Khan S, Denis P, Fan W, Kha H, Zhang J, Gong Y, Martin L, Louis JC, Yan Q, Richards WG, Citron M, Vassar R (2001). Mice deficient in BACE1, the Alzheimer’s beta-secretase, have normal phenotype and abolished beta-amyloid generation. Nat Neurosci.

[35] McKenney RJ, Huynh W, Tanenbaum ME, Bhabha G, Vale RD (2014). Activation of cytoplasmic dynein motility by dynactin-cargo adapter complexes. Science.

[36] Moechars D, Lorent K, De Strooper B, Dewachter I, Van Leuven F (1996). Expression in brain of amyloid precursor protein mutated in the alpha-secretase site causes disturbed behavior, neuronal degeneration and premature death in transgenic mice. EMBO J.

[37] Moughamian AJ, Holzbaur EL (2012). Dynactin is required for transport initiation from the distal axon. Neuron.

[38] Mullan M, Crawford F, Axelman K, Houlden H, Lilius L, Winblad B, Lannfelt L (1992). A pathogenic mutation for probable Alzheimer’s disease in the APP gene at the N-terminus of beta-amyloid. Nat Genet.

[39] O’Connor T, Sadleir KR, Maus E, Velliquette RA, Zhao J, Cole SL, Eimer WA, Hitt B, Bembinster LA, Lammich S, Lichtenthaler SF, Hebert SS, De Strooper B, Haass C, Bennett DA, Vassar R (2008). Phosphorylation of the translation initiation factor eIF2alpha increases BACE1 levels and promotes amyloidogenesis. Neuron.

[40] Oakley H, Cole SL, Logan S, Maus E, Shao P, Craft J, Guillozet-Bongaarts A, Ohno M, Disterhoft J, Van Eldik L, Berry R, Vassar R (2006). Intraneuronal beta-amyloid aggregates, neurodegeneration, and neuron loss in transgenic mice with five familial Alzheimer’s disease mutations: potential factors in amyloid plaque formation. J Neurosci.

[41] Ohno M, Cole SL, Yasvoina M, Zhao J, Citron M, Berry R, Disterhoft JF, Vassar R (2007). BACE1 gene deletion prevents neuron loss and memory deficits in 5XFAD APP/PS1 transgenic mice. Neurobiol Dis.

[42] Patel AN, Jhamandas JH (2012). Neuronal receptors as targets for the action of amyloid-beta protein (Abeta) in the brain. Expert Rev Mol Med.

[43] Perlson E, Maday S, Fu MM, Moughamian AJ, Holzbaur EL (2010). Retrograde axonal transport: pathways to cell death?. Trends Neurosci.

[44] Poon WW, Blurton-Jones M, Tu CH, Feinberg LM, Chabrier MA, Harris JW, Jeon NL, Cotman CW (2011). Beta-amyloid impairs axonal BDNF retrograde trafficking. Neurobiol Aging.

[45] Rajendran L, Honsho M, Zahn TR, Keller P, Geiger KD, Verkade P, Simons K (2006). Alzheimer’s disease beta-amyloid peptides are released in association with exosomes. Proc Natl Acad Sci USA.

[46] Rajendran L, Schneider A, Schlechtingen G, Weidlich S, Ries J, Braxmeier T, Schwille P, Schulz JB, Schroeder C, Simons M, Jennings G, Knolker HJ, Simons K (2008). Efficient inhibition of the Alzheimer’s disease beta-secretase by membrane targeting. Science.

[47] Roberson ED, Scearce-Levie K, Palop JJ, Yan F, Cheng IH, Wu T, Gerstein H, Yu GQ, Mucke L (2007). Reducing endogenous tau ameliorates amyloid beta-induced deficits in an Alzheimer’s disease mouse model. Science.

[48] Sadleir KR, Eimer WA, Kaufman RJ, Osten P, Vassar R (2014). Genetic inhibition of phosphorylation of the translation initiation factor eIF2alpha does not block Abeta-dependent elevation of BACE1 and APP levels or reduce amyloid pathology in a mouse model of Alzheimer’s disease. PLoS One.

[49] Sadleir KR, Vassar R (2012). Cdk5 protein inhibition and Abeta42 increase BACE1 protein level in primary neurons by a post-transcriptional mechanism: implications of CDK5 as a therapeutic target for Alzheimer disease. J Biol Chem.

[50] Sanchez-Varo R, Trujillo-Estrada L, Sanchez-Mejias E, Torres M, Baglietto-Vargas D, Moreno-Gonzalez I, De Castro V, Jimenez S, Ruano D, Vizuete M, Davila JC, Garcia-Verdugo JM, Jimenez AJ, Vitorica J, Gutierrez A (2012). Abnormal accumulation of autophagic vesicles correlates with axonal and synaptic pathology in young Alzheimer’s mice hippocampus. Acta Neuropathol.

[51] Selkoe DJ (1991). The molecular pathology of Alzheimer’s disease. Neuron.

[52] Shi Q, Prior M, He W, Tang X, Hu X, Yan R (2009). Reduced amyloid deposition in mice overexpressing RTN3 is adversely affected by preformed dystrophic neurites. J Neurosci.

[53] Shi Q, Prior M, Zhou X, Tang X, He W, Hu X, Yan R (2013). Preventing formation of reticulon 3 immunoreactive dystrophic neurites improves cognitive function in mice. J Neurosci.

[54] Sinha S, Anderson JP, Barbour R, Basi GS, Caccavello R, Davis D, Doan M, Dovey HF, Frigon N, Hong J, Jacobson-Croak K, Jewett N, Keim P, Knops J, Lieberburg I, Power M, Tan H, Tatsuno G, Tung J, Schenk D, Seubert P, Suomensaari SM, Wang S, Walker D, Zhao J, McConlogue L, John V (1999). Purification and cloning of amyloid precursor protein beta-secretase from human brain. Nature.

[55] Stine WB, Dahlgren KN, Krafft GA, LaDu MJ (2003). In vitro characterization of conditions for amyloid-beta peptide oligomerization and fibrillogenesis. J Biol Chem.

[56] Sun X, He G, Qing H, Zhou W, Dobie F, Cai F, Staufenbiel M, Huang LE, Song W (2006). Hypoxia facilitates Alzheimer’s disease pathogenesis by up-regulating BACE1 gene expression. Proc Natl Acad Sci USA.

[57] Tang Y, Scott DA, Das U, Edland SD, Radomski K, Koo EH, Roy S (2012). Early and selective impairments in axonal transport kinetics of synaptic cargoes induced by soluble amyloid beta-protein oligomers. Traffic.

[58] Tanzi RE (2012). The genetics of Alzheimer disease. Cold Spring Harb Perspect Med.

[59] Terry RD, Masliah E, Salmon DP, Butters N, DeTeresa R, Hill R, Hansen LA, Katzman R (1991). Physical basis of cognitive alterations in Alzheimer’s disease: synapse loss is the major correlate of cognitive impairment. Ann Neurol.

[60] Tesco G, Koh YH, Kang EL, Cameron AN, Das S, Sena-Esteves M, Hiltunen M, Yang SH, Zhong Z, Shen Y, Simpkins JW, Tanzi RE (2007). Depletion of GGA3 stabilizes BACE and enhances beta-secretase activity. Neuron.

[61] Tong Y, Zhou W, Fung V, Christensen MA, Qing H, Sun X, Song W (2005). Oxidative stress potentiates BACE1 gene expression and Abeta generation. J Neural Transm.

[62] Tsai J, Grutzendler J, Duff K, Gan WB (2004). Fibrillar amyloid deposition leads to local synaptic abnormalities and breakage of neuronal branches. Nat Neurosci.

[63] Tu S, Okamoto S, Lipton SA, Xu H (2014). Oligomeric Abeta-induced synaptic dysfunction in Alzheimer’s disease. Mol Neurodegener.

[64] Turner RS, Suzuki N, Chyung AS, Younkin SG, Lee VM (1996). Amyloids beta40 and beta42 are generated intracellularly in cultured human neurons and their secretion increases with maturation. J Biol Chem.

[65] Uryu K, Chen XH, Martinez D, Browne KD, Johnson VE, Graham DI, Lee VM, Trojanowski JQ, Smith DH (2007). Multiple proteins implicated in neurodegenerative diseases accumulate in axons after brain trauma in humans. Exp Neurol.

[66] Vassar R, Bennett BD, Babu-Khan S, Kahn S, Mendiaz EA, Denis P, Teplow DB, Ross S, Amarante P, Loeloff R, Luo Y, Fisher S, Fuller J, Edenson S, Lile J, Jarosinski MA, Biere AL, Curran E, Burgess T, Louis J-C, Collins F, Treanor J, Rogers G, Citron M (1999). Beta-secretase cleavage of Alzheimer’s amyloid precursor protein by the transmembrane aspartic protease BACE. Science.

[67] Vassar R, Kuhn PH, Haass C, Kennedy ME, Rajendran L, Wong PC, Lichtenthaler SF (2014). Function, therapeutic potential and cell biology of BACE proteases: current status and future prospects. J Neurochem.

[68] Velliquette RA, O’Connor T, Vassar R (2005). Energy inhibition elevates beta-secretase levels and activity and is potentially amyloidogenic in APP transgenic mice: possible early events in Alzheimer’s disease pathogenesis. J Neurosci.

[69] Wang WX, Rajeev BW, Stromberg AJ, Ren N, Tang G, Huang Q, Rigoutsos I, Nelson PT (2008). The expression of microRNA miR-107 decreases early in Alzheimer’s disease and may accelerate disease progression through regulation of beta-site amyloid precursor protein-cleaving enzyme 1. J Neurosci.

[70] Wang X, Perry G, Smith MA, Zhu X (2010). Amyloid-beta-derived diffusible ligands cause impaired axonal transport of mitochondria in neurons. Neurodegener Dis.

[71] Wen Y, Onyewuchi O, Yang S, Liu R, Simpkins JW (2004). Increased beta-secretase activity and expression in rats following transient cerebral ischemia. Brain Res.

[72] Wen Y, Yu WH, Maloney B, Bailey J, Ma J, Marie I, Maurin T, Wang L, Figueroa H, Herman M, Krishnamurthy P, Liu L, Planel E, Lau LF, Lahiri DK, Duff K (2008). Transcriptional regulation of beta-secretase by p25/cdk5 leads to enhanced amyloidogenic processing. Neuron.

[73] Willem M, Tahirovic S, Busche MA, Ovsepian SV, Chafai M, Kootar S, Hornburg D, Evans LD, Moore S, Daria A, Hampel H, Muller V, Giudici C, Nuscher B, Wenninger-Weinzierl A, Kremmer E, Heneka MT, Thal DR, Giedraitis V, Lannfelt L, Muller U, Livesey FJ, Meissner F, Herms J, Konnerth A, Marie H, Haass C (2015). eta-Secretase processing of APP inhibits neuronal activity in the hippocampus. Nature.

[74] Wong PC, Marszalek J, Crawford TO, Xu Z, Hsieh ST, Griffin JW, Cleveland DW (1995). Increasing neurofilament subunit NF-M expression reduces axonal NF-H, inhibits radial growth, and results in neurofilamentous accumulation in motor neurons. J Cell Biol.

[75] Wu HY, Hudry E, Hashimoto T, Kuchibhotla K, Rozkalne A, Fan Z, Spires-Jones T, Xie H, Arbel-Ornath M, Grosskreutz CL, Bacskai BJ, Hyman BT (2010). Amyloid beta induces the morphological neurodegenerative triad of spine loss, dendritic simplification, and neuritic dystrophies through calcineurin activation. J Neurosci.

[76] Xia W, Yang T, Shankar G, Smith IM, Shen Y, Walsh DM, Selkoe DJ (2009). A specific enzyme-linked immunosorbent assay for measuring beta-amyloid protein oligomers in human plasma and brain tissue of patients with Alzheimer disease. Arch Neurol.

[77] Xie H, Guan J, Borrelli LA, Xu J, Serrano-Pozo A, Bacskai BJ (2013). Mitochondrial alterations near amyloid plaques in an Alzheimer’s disease mouse model. J Neurosci.

[78] Xie H, Hou S, Jiang J, Sekutowicz M, Kelly J, Bacskai BJ (2013). Rapid cell death is preceded by amyloid plaque-mediated oxidative stress. Proc Natl Acad Sci USA.

[79] Yan R, Bienkowski MJ, Shuck ME, Miao H, Tory MC, Pauley AM, Brashier JR, Stratman NC, Mathews WR, Buhl AE, Carter DB, Tomasselli AG, Parodi LA, Heinrikson RL, Gurney ME (1999). Membrane-anchored aspartyl protease with Alzheimer’s disease beta-secretase activity. Nature.

[80] Yang LB, Lindholm K, Yan R, Citron M, Xia W, Yang XL, Beach T, Sue L, Wong P, Price D, Li R, Shen Y (2003). Elevated beta-secretase expression and enzymatic activity detected in sporadic Alzheimer disease. Nat Med.

[81] Yu WH, Cuervo AM, Kumar A, Peterhoff CM, Schmidt SD, Lee JH, Mohan PS, Mercken M, Farmery MR, Tjernberg LO, Jiang Y, Duff K, Uchiyama Y, Naslund J, Mathews PM, Cataldo AM, Nixon RA (2005). Macroautophagy—a novel Beta-amyloid peptide-generating pathway activated in Alzheimer’s disease. J Cell Biol.

[82] Zempel H, Luedtke J, Kumar Y, Biernat J, Dawson H, Mandelkow E, Mandelkow EM (2013). Amyloid-beta oligomers induce synaptic damage via Tau-dependent microtubule severing by TTLL6 and spastin. EMBO J.

[83] Zetterberg H, Blennow K (2013). Biomarker evidence for uncoupling of amyloid build-up and toxicity in Alzheimer’s disease. Alzheimers Dement.

[84] Zhang X, Zhou K, Wang R, Cui J, Lipton SA, Liao FF, Xu H, Zhang YW (2007). Hypoxia-inducible factor 1alpha (HIF-1alpha)-mediated hypoxia increases BACE1 expression and beta-amyloid generation. J Biol Chem.

[85] Zhang XM, Cai Y, Xiong K, Cai H, Luo XG, Feng JC, Clough RW, Struble RG, Patrylo PR, Yan XX (2009). Beta-secretase-1 elevation in transgenic mouse models of Alzheimer’s disease is associated with synaptic/axonal pathology and amyloidogenesis: implications for neuritic plaque development. Eur J Neurosci.

[86] Zhao J, Fu Y, Yasvoina M, Shao P, Hitt B, O’Connor T, Logan S, Maus E, Citron M, Berry R, Binder L, Vassar R (2007). Beta-site amyloid precursor protein cleaving enzyme 1 levels become elevated in neurons around amyloid plaques: implications for Alzheimer’s disease pathogenesis. J Neurosci.

[87] Zhou L, Brouwers N, Benilova I, Vandersteen A, Mercken M, Van Laere K, Van Damme P, Demedts D, Van Leuven F, Sleegers K, Broersen K, Van Broeckhoven C, Vandenberghe R, De Strooper B (2011). Amyloid precursor protein mutation E682K at the alternative beta-secretase cleavage beta’-site increases Abeta generation. EMBO Mol Med.

[88] Zong Y, Wang H, Dong W, Quan X, Zhu H, Xu Y, Huang L, Ma C, Qin C (2011). miR-29c regulates BACE1 protein expression. Brain Res.

