# Supplementary material for: Presynaptic dystrophic neurites surrounding amyloid plaques are sites of microtubule disruption, BACE1 elevation, and increased Aβ generation in Alzheimer’s disease
Source: Acta Neuropathol. 2016 Mar 18;132:235–56. doi: 10.1007/s00401-016-1558-9 (PMC4947125; doi:10.1007/s00401-016-1558-9)
Supplement: Supplementary file 1 — Supplementary material 1 (PDF 367228 kb) [file 401_2016_1558_MOESM1_ESM.pdf]

Electronic Supplementary Materials for:

Presynaptic dystrophic neurites surrounding amyloid plaques are sites of microtubule disruption, BACE1 elevation, and increased Aβ generation in Alzheimer's disease

Katherine R. Sadleir[1], Patty C. Kandalepas[1], Virginie Buggia-Prevot[2#], Daniel A. Nicholson[3], Gopal Thinakaran[2], and Robert Vassar[1]*

[1]Department of Cell and Molecular Biology, Feinberg School of Medicine, Northwestern University, Chicago, IL 60611 USA

[2] Departments of Neurobiology, Neurology, and Pathology, The University of Chicago, Chicago, IL 60637 USA

[3] Department of Neurological Sciences, Rush University

Chicago, IL 60612 USA

[#] Current address:

The Neurodegeneration Consortium

Institute for Applied Cancer Science (IACS)

The University of Texas MD Anderson Cancer Center

Houston, TX 77054 USA

*Corresponding author

r-vassar@northwestern.edu

ph: 312-503-3361

fax: 312-503-7912

Supplementary Methods:

Propidium Iodide staining and quantification of live cells:

Primary neurons were isolated, labeled with Tubulin Tracker and treated with 10μM Aβ42 oligomers or vehicle, as described in main text Methods. After 3.5 hours of Aβ42 or vehicle treatment, cells were labeled for 30 minutes with 10μg/ml propidium iodide (Molecular Probes), then imaged at 20x on a Nikon T100 Eclipse microscope for capturing representative Tubulin Tracker, propidium iodide, and DIC image frames. For quantification of live cells, four vehicle treated and four Aβ42-treated dishes were imaged, and propidium iodide and DIC image frames were merged in Photoshop. ImageJ Cell Counter was used to count propidium iodide-stained nuclei and cell bodies not containing propidium iodide in a total of 9-11 frames per condition. The percentage of live cells in each frame was averaged and Students t-test performed in Prism.

Live imaging of BACE1-YFP and NPY-mCherry trafficking in primary neurons:

All animal studies were approved by the Institutional Animal Care and Use Committee and conducted in accordance with University of Chicago Animal Care guidelines. Hippocampal neurons were cultured from E17 mouse embryos as previously described [6]. Dissociated neurons were cultured on poly-D-lysine coated glass coverslips suspended over a monolayer of primary astrocytes prepared from P0–P2 mouse pups. Cultures were maintained in Neurobasal supplemented with B27 serum-free and GlutaMAX-I supplement (Invitrogen). The C-terminally EYFP-tagged BACE1 construct has been described previously [3]. The NPY-mCherry expression vector was provided by Dr. Gary Banker. Neurons were transfected on DIV11 with Lipofectamine 2000 (Invitrogen). On DIV14, neurons were exposed to 10μM Aβ42 or vehicle for 7 hours, then used for live-cell imaging to measure BACE1-YFP and NPY-mCherry

particle motility. Live-cell images were acquired on a motorized Nikon TE 2000 microscope maintained at 37°C in a custom-designed environment chamber, at the rate of one frame per second, using 60X (NA 1.49) objective and Cascade II:512 CCD camera (Photometrics). Stacks of images were opened in ImageJ, and individual neurites traced with the segmented line tool. For each neurite traced, as indicated by the arrows along the representative neurites in the left panels of Figure S1, a kymograph was generated (shown in right panels) using the "MultipleKymograph" plugin. For each kymograph, the stationary and motile particles were counted, using the "Cell Counter" plugin in ImageJ. Motile particles were defined as puncta that exhibited lateral displacement in the kymograph. For each neurite, the percent of motile particles was calculated from these data. Each point in the scatter plot indicates a neurite and the y-axis value is the percentage of motile particles in that given neurite. Kymographs were generated from 11 neurites of vehicle-treated neurons, which contained a total of 245 NPY-mCherry and 325 BACE1-YFP particles, and from 20 Aβ42-treated neurites, which contained a total of 524 NPY-mCherry and 691 BACE1-YFP particles. We observed a significant decrease in the average percentage of motile particles per neurite for both NPY-mCherry and BACE1-YFP puncta.

Quantification of tubulin immunofluorescence in peri-plaque dystrophies:

After capturing multichannel confocal images for BACE1 (red) and tubulin isoform (green) immunofluorescence signals with NIS Elements software, the single channel tiff files were opened in ImageJ for quantification. Using the region of interest (ROI) manager, free-hand ROIs were drawn around dystrophies in the halo of high BACE1 signal around plaque cores, and the same number of similar sized ROIs was drawn in normal appearing nearby neuropil in the same image. Using the "MultiMeasure" tool, the total intensity for each ROI was measured in the BACE1

channel, and in the tubulin channel, and the ratio between the two channels determined in Excel. For human tissue 16 dystrophies and corresponding non-dystrophic regions were measured per case. For murine tissue, BACE1/tubulin isoform ratios were determined for 11-20 BACE1-positive dystrophic regions and a corresponding number of nearby normal neuropil areas. Mouse dystrophies were much more numerous and single dystrophies harder to distinguish than human dystrophies, so the whole halo of high BACE1 signal was sometimes used a single ROI for mice. BACE1/tubulin ratios for individual dystrophies or normal neuropil regions are represented by dots in the scatter plots. Mean ratios were calculated for each AD case or mouse and compared with a two-tailed t-test, with Welch correction for unequal variance in Prism software.

To generate the intensity plots shown in Fig. 3c, a two-channel BACE1 and tubulin immunofluorescence image tiff file was opened in ImageJ, and the “RGB profiles tool” macro was used to draw a line and measure fluorescence intensity in each channel for each pixel along that line (Fig. 3c, top panel). In Excel, the intensities in arbitrary units (AU) were graphed against distance along the line in microns (Fig. 3c, bottom panel).

Multiphoton confocal microscopy live imaging of BACE1-YFP transgenic mouse brain slices

BACE1-YFP transgenic mice were generated as previously described [3]. Briefly, eYFP was fused to the coding region of human BACE1, and cloned into the PMM400 tetO expression vector (gift of M. Mayford, The Scripps Institute, La Jolla, CA). This construct was used to make several tetO promoter BACE1-YFP transgenic mouse lines, which were then crossed to transgenic mice expressing the tet transactivator (tTA) from the forebrain pyramidal neuron-specific CaMKII promoter (line B, gift of M. Mayford) generating CaMKII:BACE1-YFP bigenic mice that express BACE1-YFP in forebrain

neurons (referred to as BACE1-YFP mice). Bigenic females from a high expressing BACE1-YFP mouse line (#429) were crossed to 5XFAD males to generate 5XFAD;BACE1-YFP and non-5XFAD BACE1-YFP littermates.

For live multiphoton confocal microscopy imaging, 400µm coronal slices were prepared from adult 5XFAD;BACE1-YFP mice anesthetized with ketamine/xylazine, then perfused with ice cold sucrose-ACSF solution containing: 85 mM NaCl, 2.5 mM KCl, 1.25 mM NaH_2PO_4, 25 mM $NaHCO_3$, 25 mM glucose, 75 mM sucrose, 0.5 mM $CaCl_2$, and 4 mM $MgCl_2$, equilibrated with 95% O_2/5% CO_2. After sectioning on a vibratome, slices were warmed to 28°C, and a slow exchange was made of the oxygenated sucrose-ACSF for oxygenated sodium-ACSF solution containing: 125 mM NaCl, 2.4 mM KCl, 1.2 mM NaH_2PO_4, 25 mM $NaHCO_3$, 25 mM glucose, 1 mM $CaCl_2$, and 2 mM $MgCl_2$. The slices were then maintained at room temperature in a bath perfused with aerated ACSF, and for 1 hour prior to imaging were incubated in aerated ACSF containing 1:20,000 dilution of 1mg/ml Thiazine red (Sigma). For live-cell imaging, each slice was positioned on the microscope stage, perfused with ACSF and maintained at 30°C. Slices were excited at 920 nm with Chameleon Vision titanium sapphire laser (laser range 690–1040 nm) using a Nikon A1R-MP+ multiphoton confocal microscope. Images were acquired using a 25X (NA 1.1) water immersion objective with NIS elements software. Z-stacks of images were assembled and 3D reconstructions of neuritic plaques were generated and movies made using NIS elements software.

Supplementary Text:

Dystrophic axons appear in physical contact with amyloid deposits in 3D reconstructions of live brain slices of BACE1-YFP transgenic mice

Our immunofluorescence confocal and electron microscopic analyses suggested that BACE1-positive dystrophic neurites are in close physical proximity to amyloid deposits, while normal neuropil exists a short distance way, thus implying that the dystrophy-promoting neurotoxic effects of Aβ are very short-range. To confirm this conclusion and to establish a model for the study of dystrophic neurite formation in living tissue, we generated a multi-transgenic mouse in which the 5XFAD transgenes are expressed together with transgenic BACE1-YFP fusion protein under control of the forebrain-specific CamKII promoter (5XFAD; BACE1-YFP mice). As we have described previously [12] 5XFAD; BACE1-YFP mice display BACE1-YFP accumulation in dystrophic neurites in a halo pattern around plaques similar to that of endogenous BACE1 in 5XFAD mice (Fig. S3a). We then performed multiphoton confocal microscopy imaging of live 5XFAD; BACE1-YFP brain slices co-stained with Thiazine Red to visualize amyloid deposits. The combination of the thick brain slices together with the large depth of focus inherent in mulitphoton microscopy allowed us to image normal appearing processes as they merged into peri-plaque dystrophies (Fig. S3b). Additionally, we were able to reconstruct 3-dimensional images of individual dystrophic neurites (Fig. S3c, Video S1) and whole neuritic plaques (Fig. S3d, Video S2). While many BACE1-YFP positive dystrophic neurites appeared as individual bulb-like swellings, possibly due to the very strong tendency of BACE1 to localize to presynaptic terminals [7], some appeared connected together exhibiting a “beads on a string” morphology (Fig. S3c, Video S1), reminiscent of the neuritic beading observed in Aβ42-treated primary neurons (Figs. 1 and 2). Interestingly, all BACE1-YFP positive dystrophic

neutites appeared to be in direct physical contact with amyloid deposits at some point along their length, usually at the largest part of the dystrophy, suggesting contact is necessary for dystrophy formation. These observations are consistent with our hypothesis that microtubules and BACE1 trafficking are disrupted in the very near vicinity of plaques.

The potential role of η-secretase cleavage of APP in dystrophic neurite formation

The recently discovered η-secretase processing of APP [14] produces an N-terminal soluble APP ectodomain (sAPPη) that is analogous to that generated by BACE1 (sAPPβ), however η-secretase cuts APP ~92 amino acids N-terminal to the BACE1 cleavage site in APP (Fig. S5). The membrane-bound C-terminal fragment generated by η-secretase is subsequently cut by BACE1 or α-secretase to produce Aη-β or Aη-α, respectively. For assessing BACE1 cleavage of APP in peri-plaque dystrophic neurites (Figs. 8, 9), we used the sAPPβ neoepitope antibody (ANJJ) that detects only the BACE1-cleaved free C-terminus of sAPPβ with the Swedish FAD mutation. Therefore, ANJJ does not recognize sAPPη or Aη-α, but ANJJ does not distinguish between sAPPβ and Aη-β in immunostained 5XFAD brain sections (green rectangles, Fig. S5). Note that ANJJ immunoreactivity is abolished in 5XFAD; BACE1-/- negative control mice (Fig. 8a). Despite ANJJ cross-recognition of both sAPPβ and Aη-β, the main conclusion of our study is not changed, namely that BACE1 cleavage is increased in dystrophies surrounding amyloid deposits. ANJJ immunoreactivity serves as a surrogate marker of BACE1 activity. Since we observe increased ANJJ immunostaining in peri-plaque dystrophies, we conclude that BACE1 activity is elevated in these dystrophies. In support of this conclusion, we also observe increased immunoreactivity for the free N- and C-terminal neoepitopes of Aβ, indicating that Aβ generation is also

elevated in peri-plaque dystrophies. Even though we cannot distinguish between sAPPβ and Aη-β, increased ANJJ signal indicates elevated BACE1 activity in peri-plaque dystrophies. We also note that Aη-β may be increased in BACE1-positive peri-plaque dystrophies. However, in contrast to Aη-α, Aη-β does not impair LTP or suppress neuronal activity [14]. Since Aη-α would not be expected to increase (and may actually decrease) with elevated BACE1 in dystrophic neurites, the η-secretase pathway may not have a large role in toxicity associated with neuritic dystrophy. However, a potential role for Aη in dystrophic neurite formation will be an interesting avenue to pursue in future studies.

BACE1-positive and Tau-positive dystrophic neurites are likely presynaptic and postsynaptic in origin, respectively.

Tau aggregates are present around senile plaques, mainly in the form of neuropil threads and neurofibrillary tangles consisting of paired helical filaments of Tau (reviewed in [2]). Neuropil threads and neurofibrillary tangles are likely dendrite and cell body in origin, respectively, and appear to be distinct from swollen dystrophies that appear to be axonal in nature [4,5,15]. Thus, we suggest that swollen BACE1-positive peri-plaque dystrophies are presynaptic in origin and mostly lack Tau aggregates, while dendritic dystrophies mainly exhibit Tau aggregates. This conclusion is supported by the observation in this article and others [7,18,19] that BACE1-positive dystrophies co-localize significantly with the synaptic vesicle protein synaptophysin but completely lack the somatodendritic marker MAP2 (Fig. 6, Fig. S3), and that Aβ causes Tau to mis-sort into the dendrite [16,17].

Supplementary Tables:

Table S1: List of antibodies, dilutions, and sources used in this study.

Antibody	Clone	Dilution	host	clonality	Source
BACE1	3D5	1:200	mouse	monoclonal	Vassar lab, Zhao et al. 2007
BACE1	EPR3956	1:200	rabbit	monoclonal	Abcam, #ab108394
βIII-tubulin	TuJ1	1:50	mouse	monoclonal	Dr. Lester Binder
acetylated α-tubulin	6-11B-1	1:50	mouse	monoclonal	Sigma, T7451
polyglutamylated α/β tubulin	B3	1:50	mouse	monoclonal	Sigma, T9822
dynamitin	EPR5094	1:200	rabbit	monoclonal	Abcam, #ab109752
p150glued		1:200	rabbit	polyclonal	Millipore, AB6048
dynein intermediate chain	74.1	1:200	mouse	monoclonal	Millipore, MAB1618
kinesin heavy chain	H2	1:200	mouse	monoclonal	Millipore, MAB1614
bassoon	D63B6	1:200	rabbit	monoclonal	Cell Signaling, #6897
synaptophysin	Sy38	1:300	mouse	monoclonal	Millipore, MAB5258
MAP2	AP14	1:200	mouse	monoclonal	Dr. Lester Binder
cathepsin D	EPR3057Y	1:200	rabbit	monoclonal	Abcam, #ab75852
sAPPβ (ANJJ)		1:1000	rabbit	polyclonal	Drs. Lawrence Rajendran and Patrick Keller
n-terminus of Aβ	3D6	1:600	mouse	monoclonal	Dr. Lisa McConlogue
Aβ42-specific	H31L21	1:600	rabbit	monoclonal	ThermoFisher, #700254
n-terminal APP (Karen)		1:1000	goat	polyclonal	Dr. Virginia Lee

Table S2: List of post-mortem brain samples with clinical and pathological information. Human post-mortem brain tissue (superior temporal gyrus) was obtained from 3 AD patients and 3 cognitively normal controls diagnosed at the Cognitive Neurology and Alzheimer's Disease Center with approval from the Northwestern University IRB. The case number, age, sex, Braak stage [1], CERAD score [8], and Thal amyloid phase [13] of each case are listed.

Case #	Age	Sex	Braak stage	CERAD	Thal amyloid phase
A11-193	75	female	V	freq. (C)	4
A11-78	61	female	VI	freq. (C)	5
A14-190	63	male	VI	freq. (C)	4
A01-53	64	male	0	0	0
A98-03	60	female	0	0	0
A99-106	78	female	0	0	0

Table S3: Summary of mice used for immunofluorescence staining to determine which proteins are plaque-associated and co-localize with BACE1 in dystrophies in 5XFAD mice. All antibodies were used in a co-stain with anti-BACE1 antibody.

antibody	number of mice	age/sex
Aβ42	8	5-6 mo/4 F, 2 M<br>9 mo/1 M, 12 mo/1 F
acetylated α-tubulin	5	5-6 mo/2 F, 3 M
bassoon	5	5-6 mo/2 F, 3 M
cathepsinD	6	5-6 mo/2 F, 3 M<br>12 mo/1 F
dynamitin	5	5-6 mo/2 F, 3 M
dynein int. chain	5	5-6 mo/2 F, 3 M
kinesin heavy chain	5	5-6 mo/2 F, 3 M
p150glued	5	5-6 mo/2 F, 3 M
poly-glutamylated tubulin	5	5-6 mo/2 F, 3 M
sAPPβ sw (ANJJ)	7	5-6 mo/4 F, 2 M<br>9 mo/1 M

Table S4: References for proteins for which BACE1 co-staining and plaque-associated localization have been previously published. All antibodies were used in a co-stain with anti-BACE1 antibody.

antibody	references
APP	Zhao et al., 2007; Zhang et al., 2009;<br>Kandalepas et al., 2013
Aβ (3D6)	Zhang et al., 2009
βIII-tubulin	Zhao et al., 2007; Kandalepas et al., 2013
MAP2	Zhao et al., 2007; Zhang et al., 2009;<br>Kandalepas et al., 2013
synaptophysin	Zhao et al., 2007; Zhang et al., 2009;<br>Kandalepas et al., 2013

Supplementary Figure and Video legends:

Fig. S1: Neuritic beading and microtubule disruption occur before cell death in Aβ42-treated primary neurons. Primary neuron cultures were labeled with Tubulin (Tub) Tracker (green), then exposed to 10μM Aβ42 oligomers for 3.5 hours, stained with propidium iodide (PI; red) for live cell quantification, and imaged as described in Supplementary Methods. (a) Representative PI, Tub Tracker, and DIC merged images

of primary neurons reveal that after 3.5 hours, microtubule disruption and neuritic beading (white arrowheads) are apparent in 10µM Aβ42-treated neurons, but not in vehicle-treated cells. (b) Propidium iodide images were merged with DIC images (9-11 frames per condition), as shown in (a) and the percentage of live cells was quantified. As in (a) and in Fig. 2, neuritic beading is more apparent in Aβ42-treated neurons compared to vehicle (first two panels). No significant difference in the percentage of live cells between vehicle and Aβ42-treated neurons was observed (third panel). NS = not significant.

Fig. S2: Aβ42 oligomers disrupt anterograde and retrograde trafficking of BACE1 in primary neurons. Primary murine hippocampal neurons co-transfected with BACE1-YFP and NPY-mCherry were exposed to 10µM Aβ42 oligomers (b) or vehicle (a) for 7 hours. To assess the effects of Aβ42 on the trafficking of vesicles containing BACE1-YFP or NPY-mCherry, neurons were live-imaged at 1 frame/sec for 4 minutes using a motorized Nikon TE 2000 microscope maintained at 37°C in a custom-designed environment chamber. (a and b) Shown are representative single frames of neurites imaged from vehicle-treated (a) or Aβ42-treated (b) neurons co-transfected with BACE1-YFP (green) and NPY-mCherry (red). Cell bodies are left of the frames shown, and arrows indicate neurites used to generate the kymographs shown to the right of each frame. The direction of anterograde motion is to the right and time proceeds from top to bottom in each kymograph. Scale bars = 25µm. (c) The percentage of motile BACE1-YFP or NPY-mCherry labeled particles in 11 neurites of vehicle- and 20 neurites of Aβ42-treated neurons was calculated and averaged for each condition. Each point on the scatter plot corresponds to the percentage motile particles in a given neurite. For further details on quantification, please see Supplementary Materials and Methods. Note

that motile particles were significantly decreased in neurites exposed to Aβ42 compared to vehicle. Error bars = SEM; *, $p<0.05$; **, $p<0.01$.

Fig. S3: Lower magnification images of co-stained BACE1-positive peri-plaque dystrophic neurites. Human AD (Column 1) and 5XFAD mouse (Columns 2-12) brain sections were co-stained with antibodies against BACE1 (red) and other proteins (green) and imaged by confocal microscopy as described in Figs. 3, 5, and 6. Lower magnification images are shown displaying multiple amyloid deposits for each co-stain as additional examples of the relative localization patterns of BACE1 and the respective protein, which confirm the results presented in Figs. 3, 5, and 6. Including our previously published studies, we have now analyzed BACE1 immunostaining around amyloid deposits in ~50 5XFAD mice, and we have observed that at all ages, nearly 100% of plaques have BACE1 accumulation in dystrophic neurites (e.g., see lower magnification images in [7], Fig. 4a; [12] Figs. 5&7; [11], Fig 4) that appear quite similar to each other with some minor variations in shape and size. Asterisks (*) identify plaque cores. DAPI (blue) was used to identify nuclei. Note the presence of blood vessel autofluorescence in the BACE1 channel for the human AD sample (Column 1) Scale bars = 20μm.

Fig. S4: Images of plaques in four additional mice for all antibodies whose plaque localization and co-localization with BACE1 was previously unpublished. 30μm floating sections from two 6-month old male, and two 6-month old female mice were immunostained as described in Methods with the antibodies listed in Table S3 along with either anti-BACE1 mouse monoclonal 3D5 or anti-BACE1 rabbit monoclonal (EPR3956) at the concentrations listed in Table S1. Immunostaining for BACE1 is shown in red, other proteins in green, and DAPI stain for nuclei is shown in blue. Plaque core autofluorescence in blue. All images were collected on Nikon A1 confocal microscope

with 60x objective using NIS Elements software. (a) Low magnification images of cortical layer 5 from each mouse. Scale bars = 20μm (b) High magnification images of individual plaques in cortical layer 5 from each mouse. Scale bars = 10μm. (c) Images of layer 5 cortex of secondary antibody-only control for donkey anti-mouse Alexa 488 with donkey anti-rabbit Alexa 568. Scale bars = 20μm. (d) Images of layer 5 cortex of secondary antibody-only control for donkey anti-rabbit Alexa488 with donkey anti-mouse Alexa 568. Scale bars = 20μm. Mice were not perfused before brain harvest so blood vessels show background fluorescence in some sections.

Fig. S5: BACE1-YFP positive dystrophic neurites in live brain slices have complex morphologies and are in close physical association with amyloid deposits. Multi-photon confocal microscopy of live brain slices from 5XFAD; BACE1-YFP transgenic mice was performed to assess the 3-dimentional (3D) morphologies of peri-plaque dystrophic neurites and the physical associations between plaques and dystrophies. Brain slices were incubated in Thiazine Red to visualize amyloid deposits (asterisks). (a, b) Representative multi-photon confocal images (a, single; b, Z-projection) of amyloid deposits and associated BACE1-YFP positive dystrophies in live brain slices. Swollen dystrophic neurites that surrounded amyloid deposits (red) accumulated BACE1-YFP (green) in a pattern similar to that of endogenous BACE1 (e.g., compare to Fig. 3, 5, 6). BACE1-YFP positive dystrophies generally appeared spherical or elliptical, although some with optimal orientations exhibited highly elongated shapes (b) that appeared to be continuous with axons. (c, d) 3D reconstructions of multiphoton confocal Z-projection images revealed live BACE1-YFP positive dystrophies having long extensions with varicosities exhibiting “beads on a string” morphologies (arrowheads) ending in enlarged bulbs (arrows), reminiscent of live primary neurons treated with Aβ42 (e.g., compare to Figs. 1 and 2). BACE1-YFP positive dystrophies were always in close physical

association with amyloid deposits. Inset in (d) shows a higher resolution single confocal image of the elongated BACE1-YFP positive dystrophy in the 3D reconstruction. Together, these results suggest that direct physical contact with the amyloid plaque may cause dystrophy formation. Scale bars = 5μm in all frames.

Fig. S6: Working hypothesis depicting Aβ-induced microtubule disruption, impaired axonal transport, and dystrophic axon formation. (Left) Our *in vitro* and *in vivo* results suggest a modified amyloid cascade hypothesis in which Aβ accumulation induces microtubule disruption, motor mis-localization, and impaired transport leading to axonal dystrophy and the accumulation of BACE1, APP, and increased Aβ generation in peri-plaque dystrophies that further accelerates amyloid deposition in a feed-forward mechanism. (Right top) Depicted is a normal neuron with an axon that is near but not in physical contact with a growing amyloid nidus. Microtubules (lines in the axon) are intact and support normal axonal transport of vesicles and organelles (colored circles). (Right middle) As the amyloid deposit grows and comes in close proximity to the axon, the high local concentrations of toxic Aβ species leads to localized microtubule destabilization and/or depolymerization, thus impairing axonal transport. Motor proteins and transported vesicles detach from the ends of microtubules and begin to accumulate causing the axon to swell. (Right bottom) As the amyloid deposit grows into a neuritic plaque, local microtubule disruption becomes severe further hampering transport and causing large accumulations of vesicles and organelles in axonal swellings near the plaque. Processes that depend on microtubule-based transport, such as lysosomal maturation and autophagy, are inhibited. As the plaque continues to grow, it will contact a larger number of surrounding axons, causing increasing numbers of dystrophies and neurons with impaired transport, contributing to the synaptic defects, neurodegeneration, and cognitive impairment characteristic of AD.

Fig. S7: η-secretase and amyloidogenic APP processing pathways.

In this diagram, adapted from [14], we show that the ANJJ antibody [9,10], which recognizes the free C-terminal neoepitope of sAPPβ (green vertical line) created by BACE1 cleavage of APP carrying the Swedish mutation, will detect Aη-β and sAPPβ (green rectangles) but not sAPPη or Aη-α. Since ANJJ detects only BACE1 cleaved fragments, it is an effective surrogate marker of BACE1/β-secretase activity.

Supplementary Videos:

Video S1 and S2: Movies of 3D reconstructions of BACE1-YFP positive dystrophic neurites. Live 5XFAD; BACE1-YFP brain slices were imaged by multiphoton confocal microscopy as described in Fig. S5 legend and movies of 3D reconstructions were generated of two amyloid deposits and associated BACE1-YFP positive dystrophic neurites shown in Figs. S5c and S5d. Amyloid deposits are red and BACE1-YFP positive dystrophic neurites are green.

Fig. S1: Neuritic beading occurs before cell toxicity in Aβ42 treated neurons

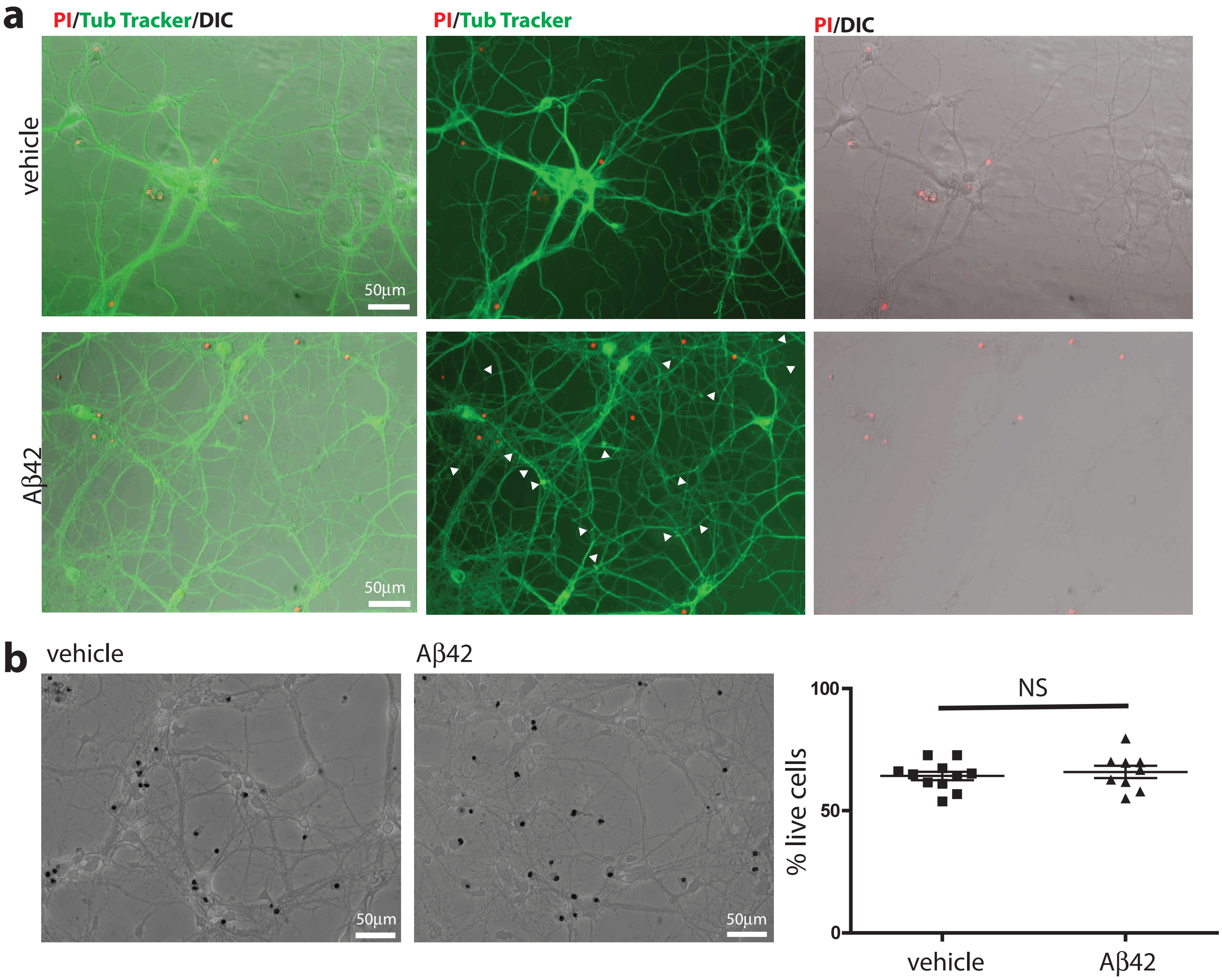

Figure S2: Aβ42 oligomers disrupt anterograde and retrograde trafficking of BACE1 in primary neurons

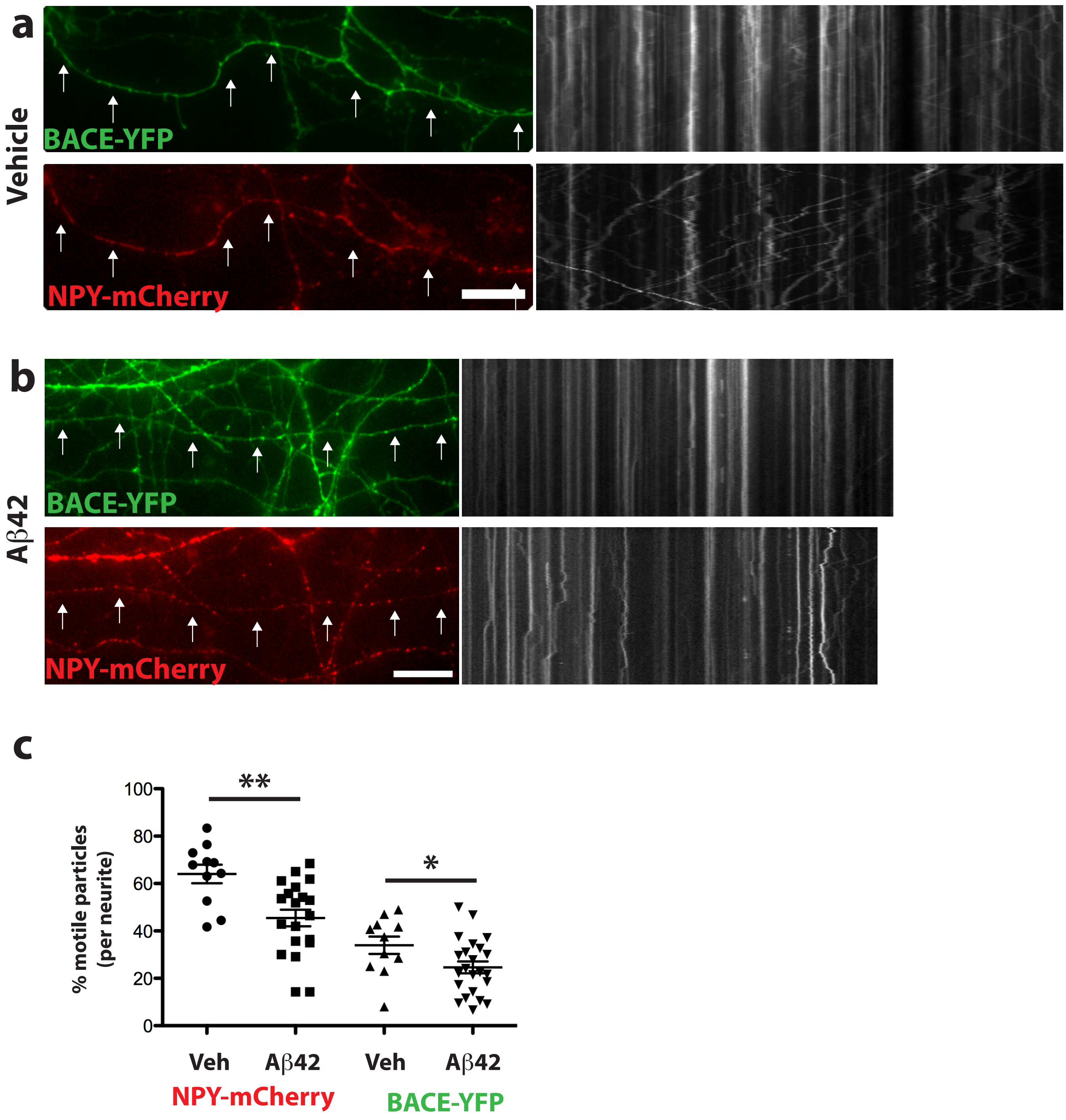

Figure S3: Lower magnification images of co-stained BACE1-positive peri-plaque dystrophic neurites

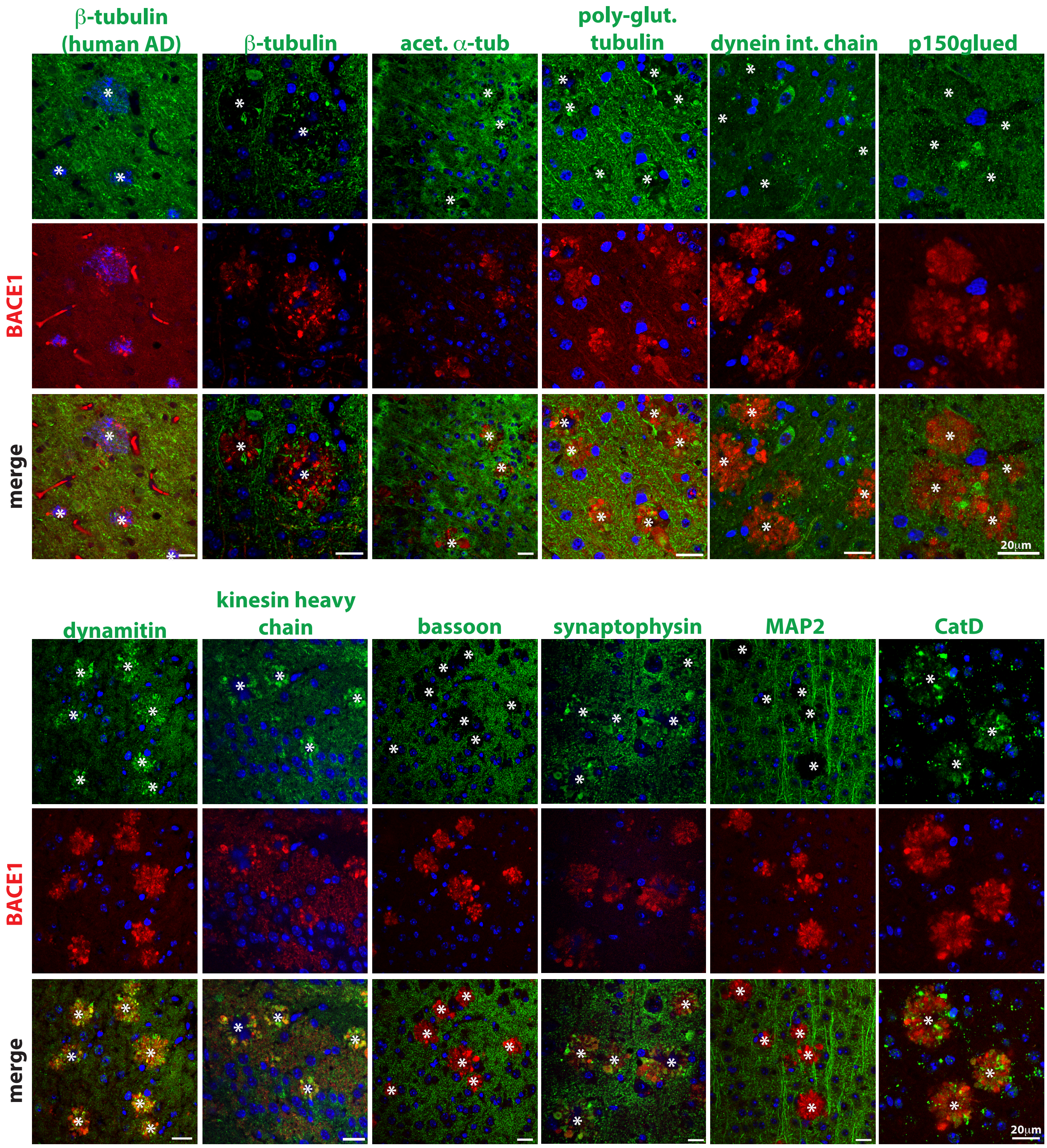

Figure S4: Images of plaques in four additional mice for all antibodies whose plaque localization, and co-localization with BACE1 was previously unpublished

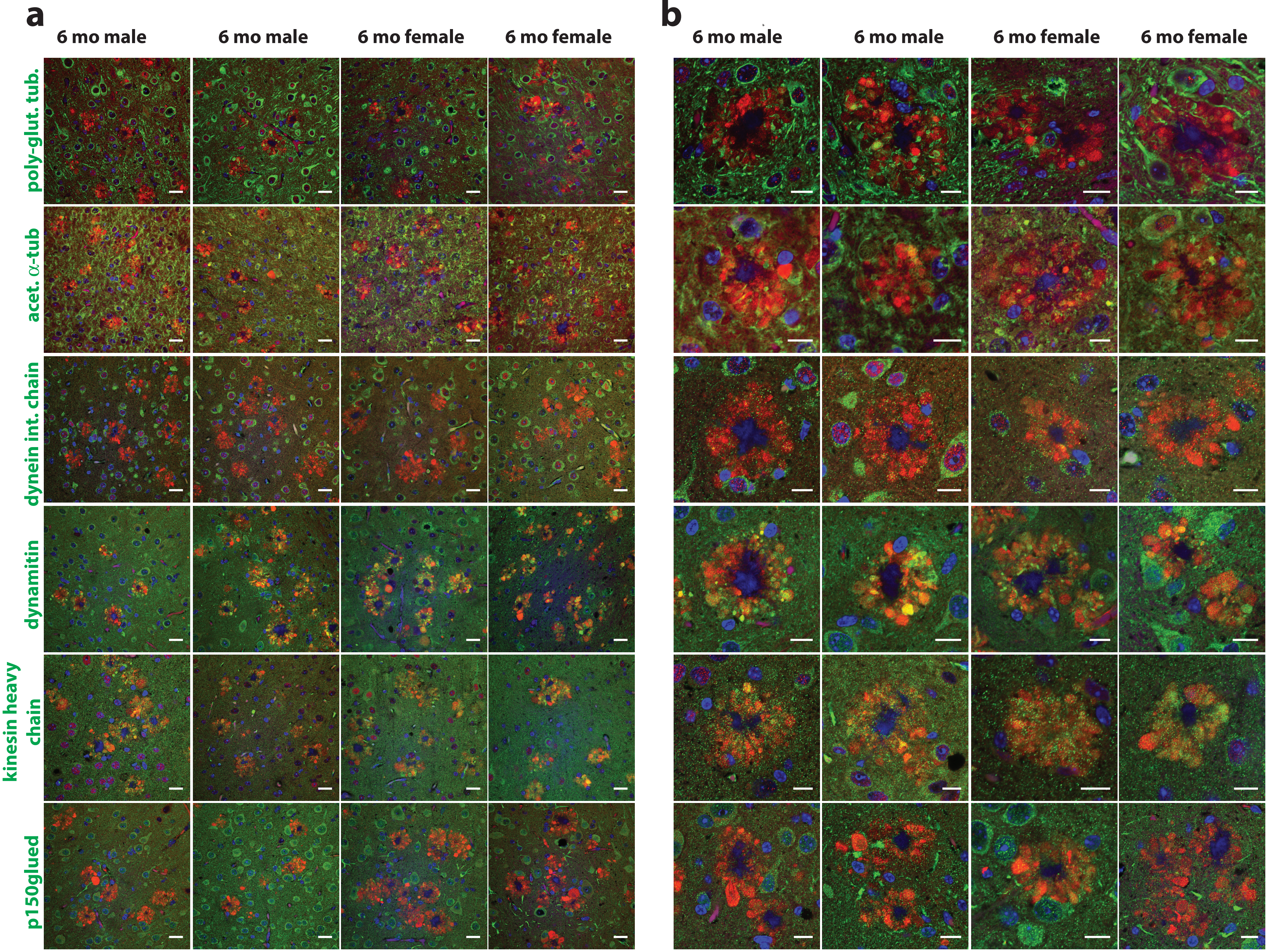

Figure S4 continued: Images of plaques in four additional mice for all antibodies whose plaque localization, and co-localization with BACE1 was previously unpublished

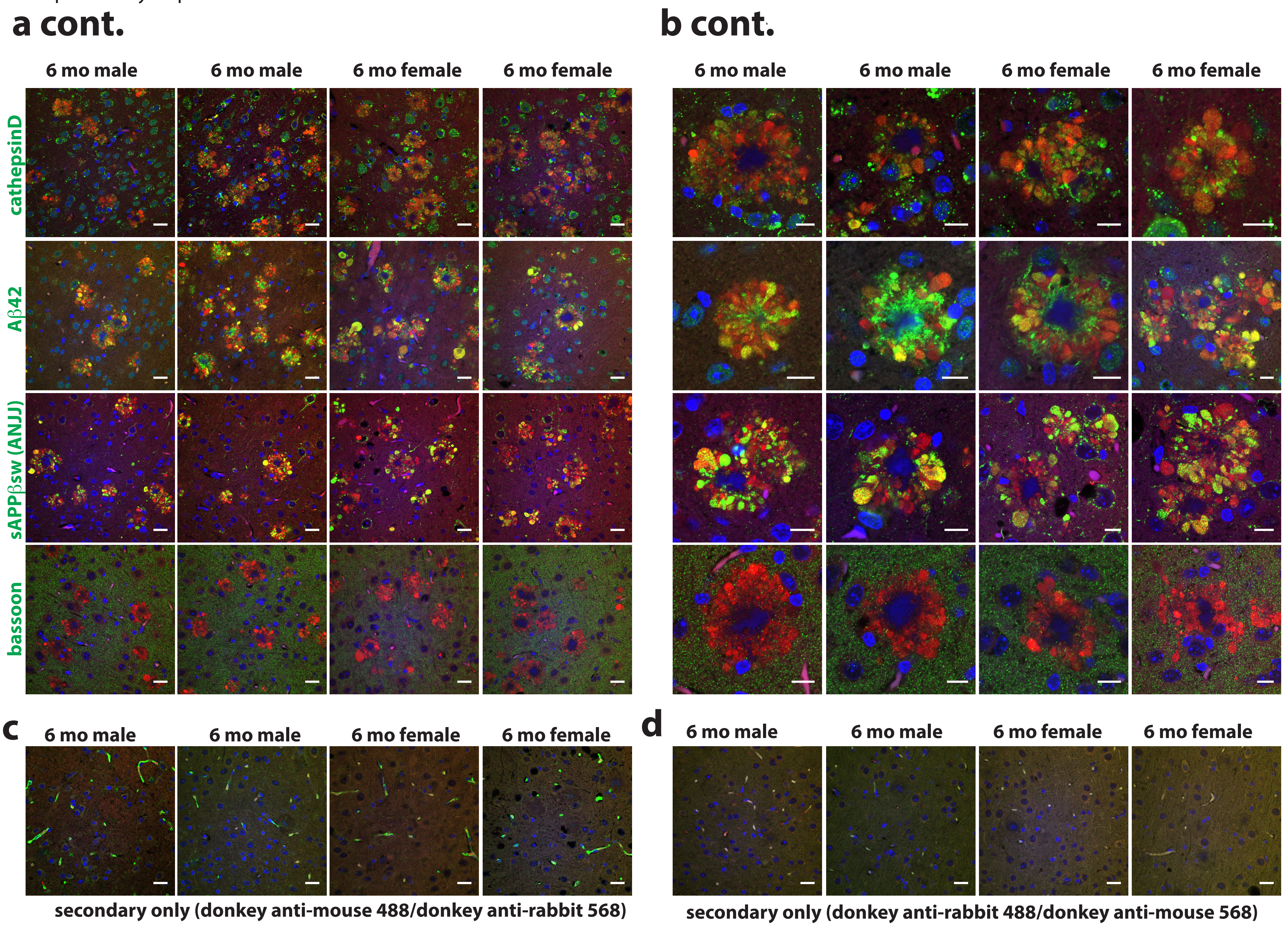

Figure S5: BACE1-YFP positive dystrophic neurites in live brain slices have complex morphologies and are in close physical association with amyloid deposits

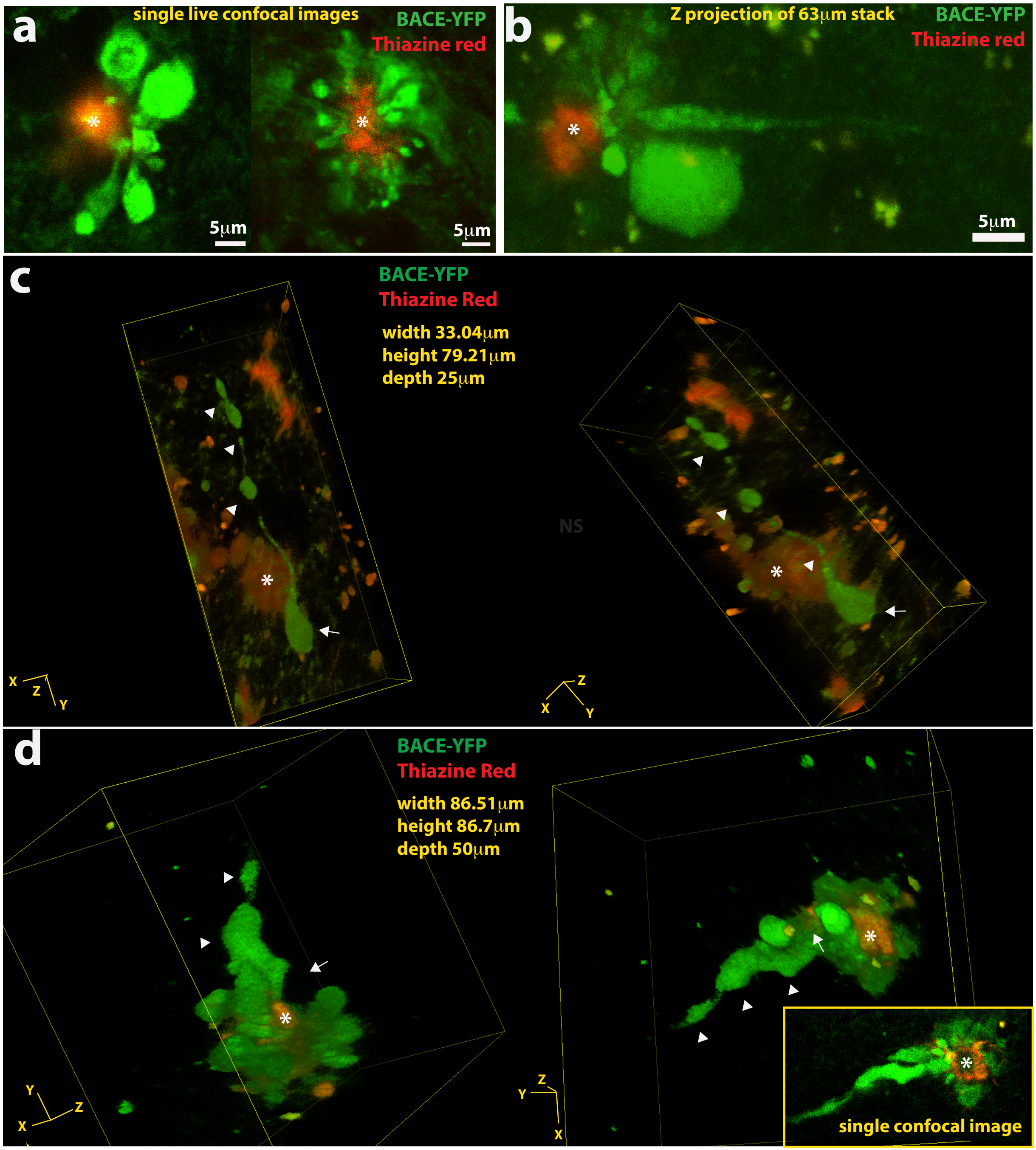

Figure S6: Working hypothesis depicting Aβ-induced microtubule disruption, impaired axonal transport, and dystrophic axon formation

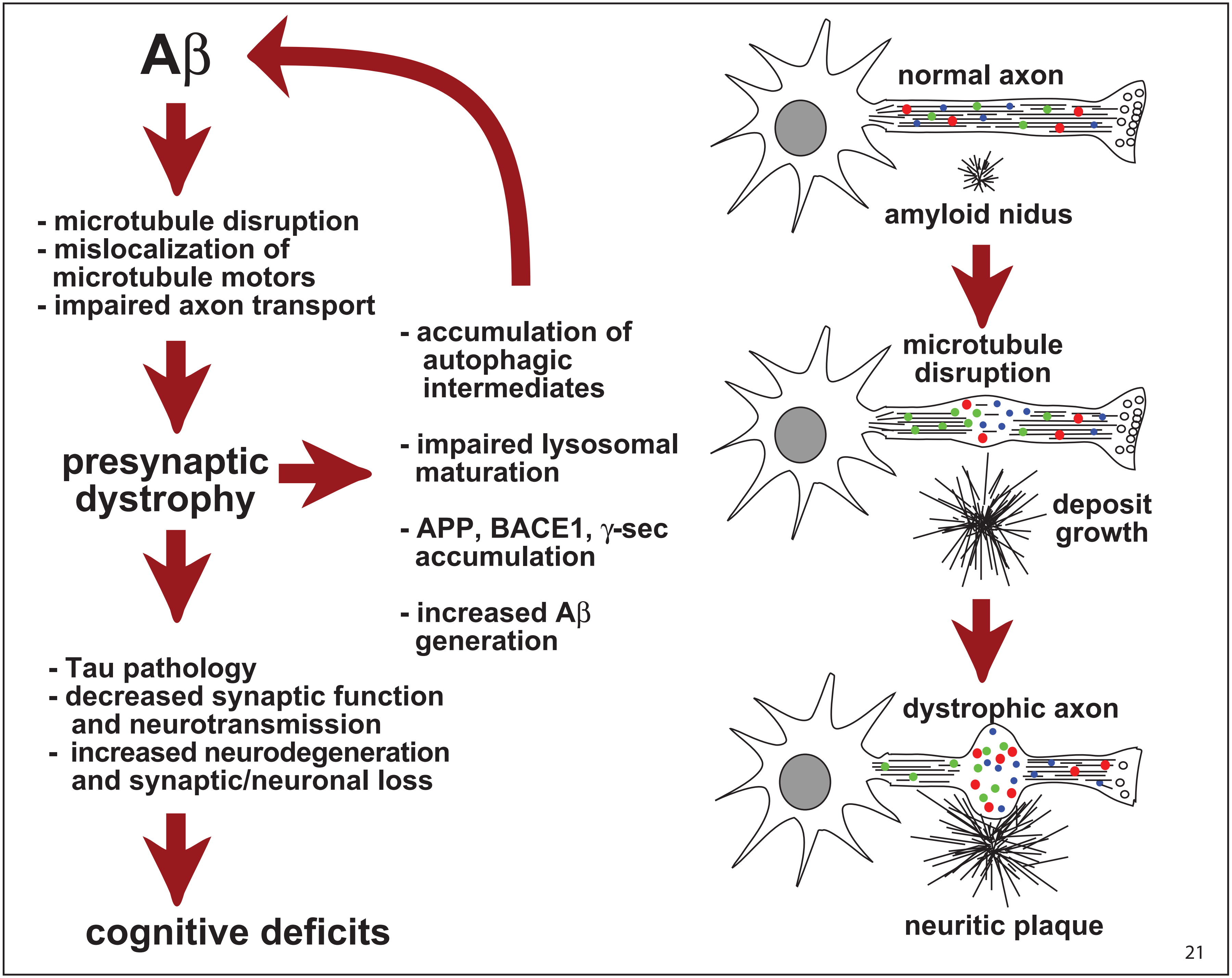

Figure S7: η-secretase and amyloidogenic APP processing pathways.

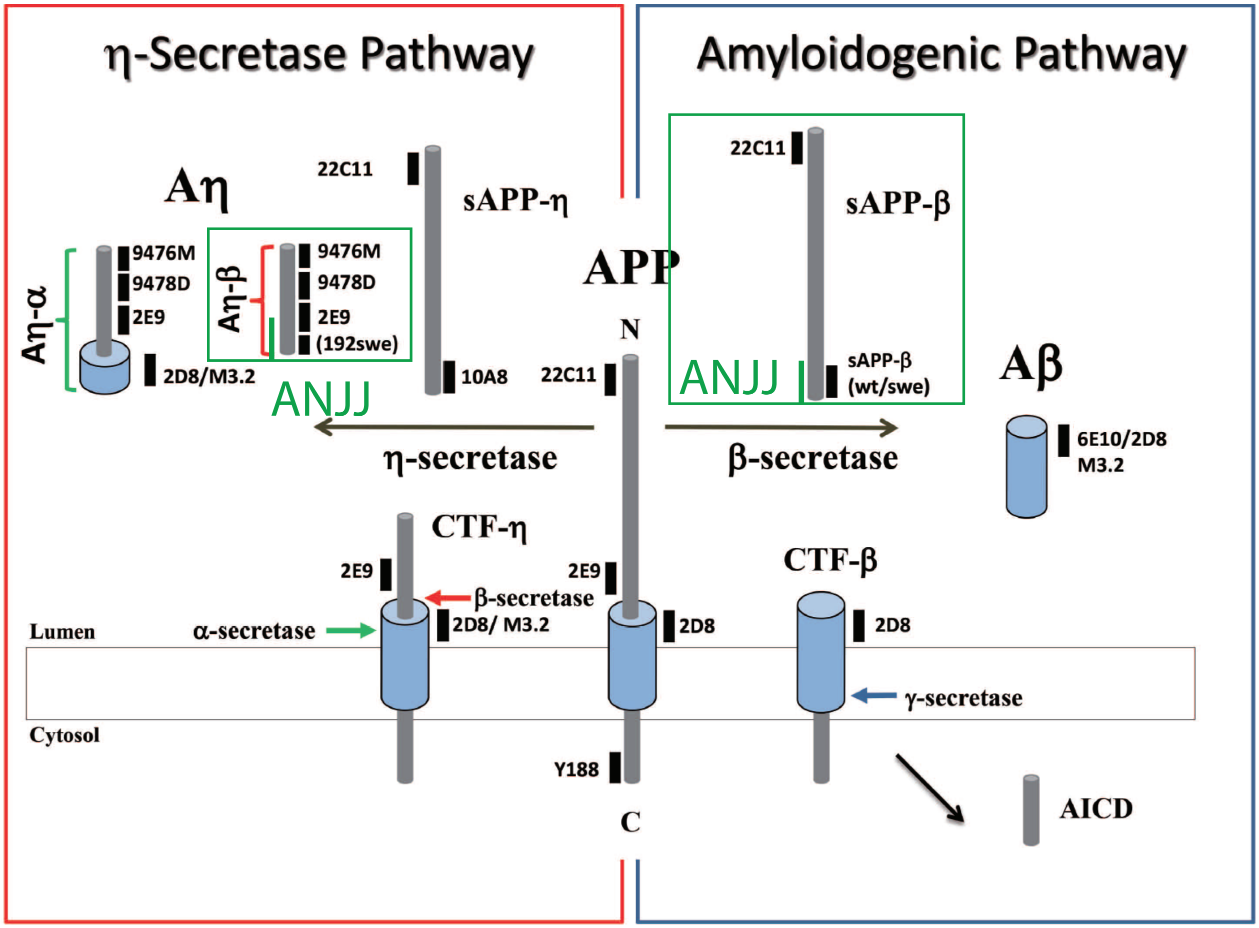

Supplementary References:

1. Braak H, Braak E (1991) Neuropathological stageing of Alzheimer-related changes. Acta neuropathologica 82:239-259
2. Brunden KR, Trojanowski JQ, Lee VM (2009) Advances in tau-focused drug discovery for Alzheimer's disease and related tauopathies. Nat Rev Drug Discov 8:783-793. doi:10.1038/nrd2959
3. Buggia-Prevot V, Fernandez CG, Riordan S, Vetrivel KS, Roseman J, Waters J, Bindokas VP, Vassar R, Thinakaran G (2014) Axonal BACE1 dynamics and targeting in hippocampal neurons: a role for Rab11 GTPase. Molecular neurodegeneration 9:1. doi:10.1186/1750-1326-9-1
4. Cras P, Kawai M, Lowery D, Gonzalez-DeWhitt P, Greenberg B, Perry G (1991) Senile plaque neurites in Alzheimer disease accumulate amyloid precursor protein. Proc Natl Acad Sci U S A 88:7552-7556
5. Dickson TC, King CE, McCormack GH, Vickers JC (1999) Neurochemical diversity of dystrophic neurites in the early and late stages of Alzheimer's disease. Exp Neurol 156:100-110. doi:10.1006/exnr.1998.7010
6. Kaech S, Banker G (2006) Culturing hippocampal neurons. Nat Protoc 1:2406-2415. doi:10.1038/nprot.2006.356
7. Kandalepas PC, Sadleir KR, Eimer WA, Zhao J, Nicholson DA, Vassar R (2013) The Alzheimer's beta-secretase BACE1 localizes to normal presynaptic terminals and to dystrophic presynaptic terminals surrounding amyloid plaques. Acta neuropathologica 126:329-352. doi:10.1007/s00401-013-1152-3
8. Mirra SS, Heyman A, McKeel D, Sumi SM, Crain BJ, Brownlee LM, Vogel FS, Hughes JP, van Belle G, Berg L (1991) The Consortium to Establish a Registry for Alzheimer's Disease (CERAD). Part II. Standardization of the neuropathologic assessment of Alzheimer's disease. Neurology 41:479-486
9. Rajendran L, Honsho M, Zahn TR, Keller P, Geiger KD, Verkade P, Simons K (2006) Alzheimer's disease beta-amyloid peptides are released in association with exosomes. Proc Natl Acad Sci U S A 103:11172-11177. doi:10.1073/pnas.0603838103
10. Rajendran L, Schneider A, Schlechtingen G, Weidlich S, Ries J, Braxmeier T, Schwille P, Schulz JB, Schroeder C, Simons M, Jennings G, Knolker HJ, Simons K (2008) Efficient inhibition of the Alzheimer's disease beta-secretase by membrane targeting. Science 320:520-523. doi:10.1126/science.1156609
11. Sadleir KR, Eimer WA, Cole SL, Vassar R (2015) Abeta reduction in BACE1 heterozygous null 5XFAD mice is associated with transgenic APP level. Molecular neurodegeneration 10:1. doi:10.1186/1750-1326-10-1
12. Sadleir KR, Eimer WA, Kaufman RJ, Osten P, Vassar R (2014) Genetic inhibition of phosphorylation of the translation initiation factor eIF2alpha does not block Abeta-dependent elevation of BACE1 and APP levels or reduce amyloid pathology in a mouse model of Alzheimer's disease. PloS one 9:e101643. doi:10.1371/journal.pone.0101643
13. Thal DR, Rub U, Orantes M, Braak H (2002) Phases of A beta-deposition in the human brain and its relevance for the development of AD. Neurology 58:1791-1800
14. Willem M, Tahirovic S, Busche MA, Ovsepian SV, Chafai M, Kootar S, Hornburg D, Evans LD, Moore S, Daria A, Hampel H, Muller V, Giudici C, Nuscher B, Wenninger-Weinzierl A, Kremmer E, Heneka MT, Thal DR, Giedraitis V, Lannfelt L, Muller U, Livesey FJ, Meissner F, Herms J, Konnerth A, Marie H, Haass C

(2015) eta-Secretase processing of APP inhibits neuronal activity in the hippocampus. Nature 526:443-447. doi:10.1038/nature14864
15. Yamaguchi H, Nakazato Y, Shoji M, Ihara Y, Hirai S (1990) Ultrastructure of the neuropil threads in the Alzheimer brain: their dendritic origin and accumulation in the senile plaques. Acta neuropathologica 80:368-374
16. Zempel H, Luedtke J, Kumar Y, Biernat J, Dawson H, Mandelkow E, Mandelkow EM (2013) Amyloid-beta oligomers induce synaptic damage via Tau-dependent microtubule severing by TTLL6 and spastin. The EMBO journal 32:2920-2937. doi:10.1038/emboj.2013.207
17. Zempel H, Thies E, Mandelkow E, Mandelkow EM (2010) Abeta oligomers cause localized Ca(2+) elevation, missorting of endogenous Tau into dendrites, Tau phosphorylation, and destruction of microtubules and spines. J Neurosci 30:11938-11950. doi:10.1523/JNEUROSCI.2357-10.2010
18. Zhang XM, Cai Y, Xiong K, Cai H, Luo XG, Feng JC, Clough RW, Struble RG, Patrylo PR, Yan XX (2009) Beta-secretase-1 elevation in transgenic mouse models of Alzheimer's disease is associated with synaptic/axonal pathology and amyloidogenesis: implications for neuritic plaque development. The European journal of neuroscience 30:2271-2283. doi:10.1111/j.1460-9568.2009.07017.x
19. Zhao J, Fu Y, Yasvoina M, Shao P, Hitt B, O'Connor T, Logan S, Maus E, Citron M, Berry R, Binder L, Vassar R (2007) Beta-site amyloid precursor protein cleaving enzyme 1 levels become elevated in neurons around amyloid plaques: implications for Alzheimer's disease pathogenesis. J Neurosci 27:3639-3649
